# Crystal structures, Hirshfeld atom refinements and Hirshfeld surface analyses of tris­(4,5-di­hydro­furan-2-yl)methyl­silane and tris­(4,5-di­hydro­furan-2-yl)phenyl­silane

**DOI:** 10.1107/S2056989020011470

**Published:** 2020-08-28

**Authors:** Anna Krupp, Eva Rebecca Barth, Rana Seymen, Carsten Strohmann

**Affiliations:** a Technische Universität Dortmund, Fakultät Chemie und Chemische Biologie, Otto-Hahn-Strasse 6, 44227 Dortmund, Germany

**Keywords:** crystal structure, di­hydro­furanyl group (DHF), Hirshfeld atom refinement (HAR), Hirshfeld surface analysis, C—H⋯O hydrogen bonds

## Abstract

The title compounds, C_13_H_18_O_3_Si (**1**) and C_18_H_20_O_3_Si (**2**), represent functional­izable di­hydro­furan­ylsilanes, which permit substitution by a variety of nucleophiles. The crystal structures of **1** and **2** display weak inter­molecular C—H⋯O hydrogen-bonding inter­actions (qu­anti­fied by Hirshfeld surface analysis), leading to a two-dimensional supra­molecular network for **1** and a one-dimensional supra­molecular network for **2**. The crystal structures of **1** and **2** were refined both on the basis of the independent atom model (IAM) and the Hirshfeld atom refinement (HAR) approach, and the results are comparatively discussed.

## Abstract 2

The crystal structures of tris­(4,5-di­hydro­furan-2-yl)methyl­silane (**1**) and -phenyl­silane (**2**) display weak inter­molecular C—H⋯O hydrogen-bonding inter­actions, which were analysed using Hirshfeld surface analysis. Futhermore, the crystal structures of (**1**) and (**2**) were refined using independent atom model (IAM) and Hirshfeld atom refinement (HAR) approaches

## Chemical context   

Tris(4,5-di­hydro­furan-2-yl)methyl­silane (**1**) and -phenyl­silane (**2**) are inter­esting starting materials for the selective synthesis of functionalized organosilanes in mol­ecular chemistry.
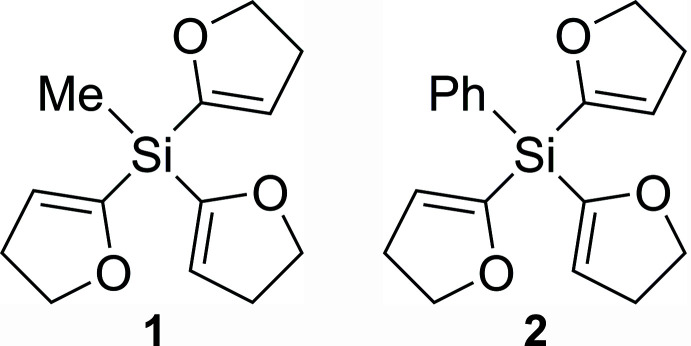


In the 1980s, Lukevits and co-workers first introduced the di­hydro­furanyl group (DHF) as a substitutable silicon-carbon leaving group (Gevorgyan *et al.*, 1989 ▸). The DHF group allows substitution by a number of nucleophiles including hydrides, li­thia­ted amides, lithium alkyls and alcohols (Lukevits *et al.*, 1993 ▸). Multiple nucleophilic substitutions using chloro­silanes show high reactivity and low selectivity. In general, the Si—O bond shows high reactivity and selectivity compared to the less or even non-reactive Si—C bond. Nonetheless, the DHF group shows a significant increase in reactivity and selectivity in the bond cleavage of Si—C bonds, which can extend the selectivity profile of functionalized organosilanes (Koller *et al.*, 2017 ▸). Furthermore, the pre-coordination by a meth­oxy group plays an important role in the control of reactions with metal-containing nucleophiles and leads to the question of whether this also applies to the DHF group (Barth *et al.*, 2019 ▸). In order to understand the coordination possibilities, the alignment of the di­hydro­furanyl group and thus the arrangement of the oxygen atoms in the crystal structure are inter­esting. In this context, we here report the crystal structures of **1** and **2**, both refined on basis of the independent atom model (IAM) and a Hirshfeld atom refinement (HAR) approach.

## Structural commentary   

**Figure 1:**
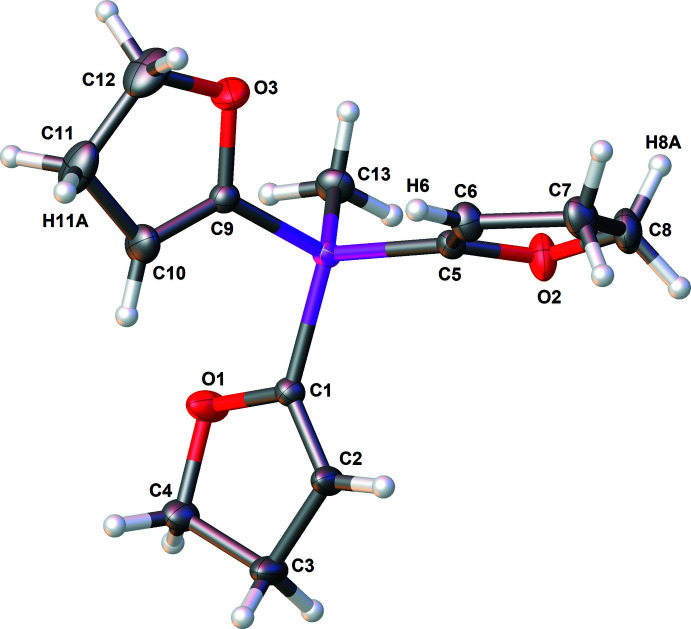
The mol­ecular structure of compound **1** with displacement ellipsoids drawn at the 50% probability level.

The mol­ecular structure of compound **1** is illustrated in Fig. 1 ▸, and selected bond lengths and angles using the results of IAM and HAR refinements are given in Table 1 ▸. In the mol­ecule of **1**, the Si—C bond lengths of the silicon–DHF groups are in a typical range and slightly longer than the silicon–methyl bond length. However, all Si—C bonds are as expected (Allen *et al.*, 1987 ▸). The silicon atom in **1** has a slightly distorted tetra­hedral environment, as shown by the deviation of the C—Si—C angles from the ideal value of 109.47°. This flexibility is often observed for Si—C single bonds (Otte *et al.*, 2017 ▸; Glidewell & Sheldrick, 1971 ▸; Kückmann *et al.*, 2005 ▸). The length of each of the C=C double bonds of the DHF groups (C1=C2, C5=C6, C9=C10) also corresponds well with the literature (Allen *et al.*, 1987 ▸).

**Figure 2:**
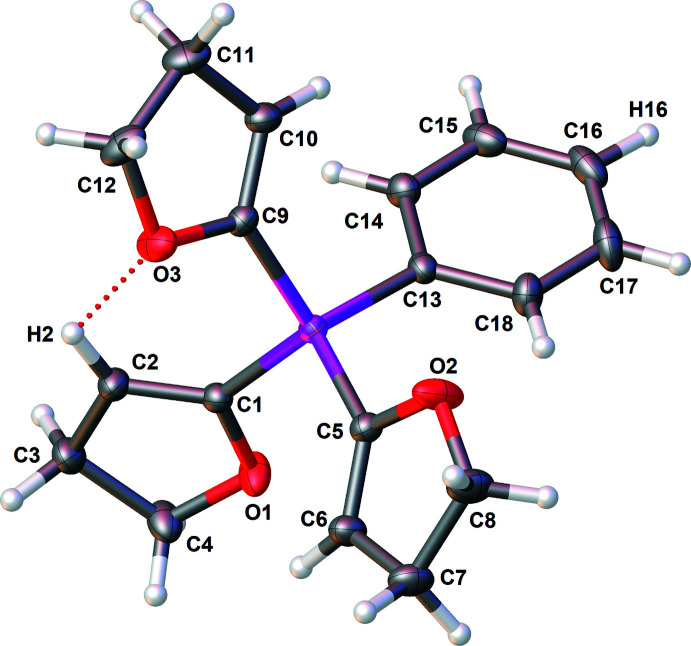
The mol­ecular structure of compound **2** with displacement ellipsoids drawn at the 50% probability level.

The mol­ecular structure of compound **2** is depicted in Fig. 2 ▸, and selected bond lengths and angles using the results of IAM and HAR refinements are collated in Table 2 ▸. The Si—C bond lengths and angles in the mol­ecule of **2** differ only marginally from those of **1**. In **2**, there is a weak intra­molecular C2—H2⋯O3 hydrogen-bonding inter­action between the H2 atom of the C1=C2 group of one DHF mol­ecule and the O3 atom of a neighbouring DHF group (Table 4 ▸), leading to a graph-set motif 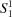
(6) (Etter *et al.*, 1990 ▸).

The Si—C bond lengths and C—Si—C angles of the IAM and HAR refinements coincide well. Slight deviations in the C=C double bond of the DHF group can be observed and the trend shows that the double bonds from HAR refinement are slightly longer.

## Hirshfeld atom refinements   

The independent atom model (IAM) approach for crystal-structure refinement cannot reliably model bonding electrons or any distortion of the electron density. An approach that takes this into consideration is Hirshfeld atom refinement (HAR), which uses aspherical atomic scattering factors calculated from tailor-made *ab initio* quantum-mechanical electron densities. This approach allows for an accurate localization of hydrogen atoms, bonding electrons and an anisotropic refinement of hydrogen atoms (Jayatilaka & Dittrich, 2008 ▸; Capelli *et al.*, 2014 ▸).

In previous (unpublished) structure refinements of compounds with di­hydro­furanyl rings performed by our group, we observed slight disorders of the oxygen atom and the methine atom of the di­hydro­furanyl ring. Therefore, results of HARs for such compounds are inter­esting in order to draw conclusions about the residual electron densities to exclude and/or model disorder. For **1** and **2**, the minimum and maximum values of residual electron density are significantly lower than those of IAM results (**1**: IAM Δ*ρ*_min_ = −0.21 e Å^−3^, Δ*ρ*_max_ = 0.55 e Å^−3^; HAR Δ*ρ*_min,max_ = ±0.21 e Å^−3^; **2**: IAM Δ*ρ*_min_ = −0.23 e Å^−3^, Δ*ρ*_max_ = 0.47 e Å^−3^; HAR Δ*ρ*_min_ = −0.17 e Å^−3^, Δ*ρ*_max_ = 0.26 e Å^−3^). In all cases, the residual densities do not indicate any disorder. For compound **1**, the residual electron density on the basis of the HAR refinement is close to O1 and H8*A* and for **2** is near C15 and H3*B*. Another aim of the Hirshfeld atom refinement was the accurate localization of hydrogen atoms. From a comparison of the C—H bond lengths of the methine groups using IAM and HAR approaches, it can be clearly observed that the C—H bonds of the HAR model are significantly longer than those of the AIM model (Table 5 ▸). Woińska *et al.* (2016 ▸) have already reported that the positions of hydrogen atoms and their corresponding bond lengths show a significantly improved agreement with neutron diffraction by refinement with HAR.

When using HAR, an improved *R*_1_ value of 0.023 was observed for compound **1**, compared to the refinement using IAM with an *R*_1_ value of 0.035 (compound **2**: *R*_1_ for HAR = 0.024 *versus* IAM = 0.037).

## Hirshfeld analyses and supra­molecular features   

**Figure 3:**
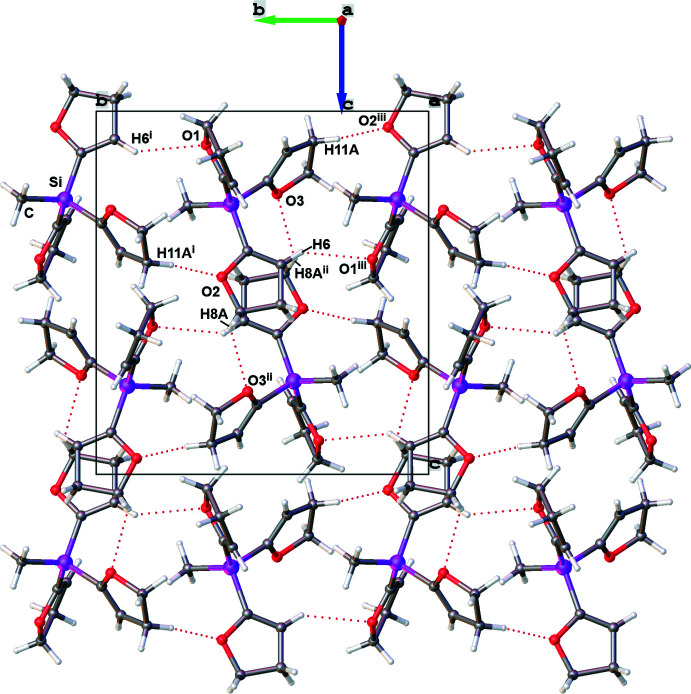
The crystal packing of compound **1** in a view along the *a* axis. C—H⋯O hydrogen bonds are shown as dashed lines. [Symmetry codes: (i) −*x* + 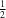, *y* + 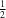, −*z* + 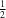; (ii) −*x*, −*y* + 1, −*z* + 1; (iii) −*x* + 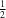, *y* − 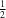, −*z* + 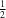].

**Figure 4:**
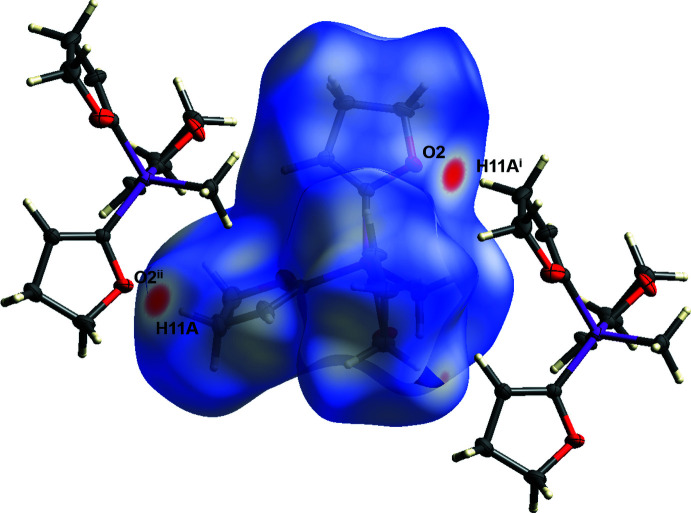
Hirshfeld surface analysis of **1** showing close contacts in the crystal. The weak hydrogen bond between oxygen atom O2 and the H11*A* hydrogen atom is labelled. [Symmetry codes: (i) −*x* + 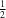, *y* + 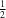, −*z* + 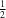; (ii) −*x* + 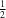, *y* − 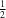, −*z* + 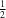].

**Figure 5:**
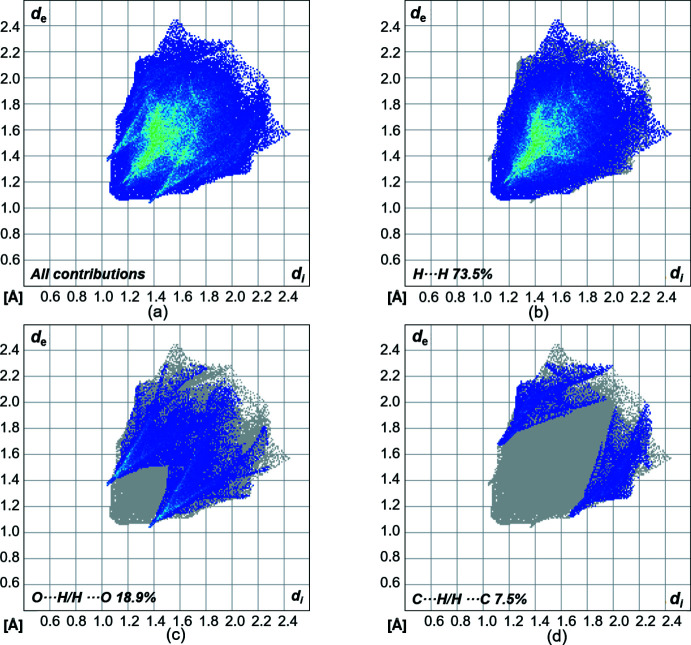
(*a*) Two-dimensional fingerprint plots for compound **1**, showing all contributions (*a*), and delineated (*b*)–(*d*) showing the contributions of atoms within specific inter­acting pairs (blue areas).

In the crystal of compound **1**, the mol­ecules are linked by a number of C—H⋯O hydrogen bonds, forming a network along the [012] direction (Fig. 3 ▸, Table 3 ▸). Considering the C⋯O distances, the strength of the hydrogen bonds can be classified as weak according to Desiraju & Steiner (1999 ▸). Hydrogen bonds C6—H6⋯O1^i^ and C11—H11*A*⋯O2^i^ lead to the formation of chains described by the graph-set motifs 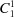
(6) and 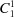
(7), respectively. The third hydrogen bond, C8—H8*A*⋯O3^ii^, leads to rings with graph-set motif 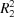
(14) (Etter *et al.*, 1990 ▸). For the C11—H11*A*⋯O2^i^ hydrogen bond, a significant inter­action can be visualized using Hirshfeld surface analysis (Spackman & Jayatilaka, 2009 ▸) generated by *CrystalExplorer* (Turner *et al.*, 2017 ▸), here indicated by the red spots (Fig. 4 ▸). The Hirshfeld surface mapped over *d*_norm_ is in the range from −0.1450 to 1.0518 a.u. The contributions of different types of inter­molecular inter­actions for **1** are shown in the two-dimensional fingerprint plots (McKinnon *et al.*, 2007 ▸) in Fig. 5 ▸. On the Hirshfeld surface, the weak van der Waals H⋯H contacts appear in the largest region (73.5% contribution). The fingerprint plot for the O⋯H/H⋯O (18.9%) inter­actions shows sharp spikes, which highlight the hydrogen bond between two mol­ecules. The C⋯H/H⋯C (7.5%) inter­actions also appear as two spikes. In summary, H⋯H, C⋯H/H⋯C and especially O⋯H/H⋯O are significant contributors, suggesting the relevance of these contacts in the packing arrangement of the crystal structure.

**Figure 6:**
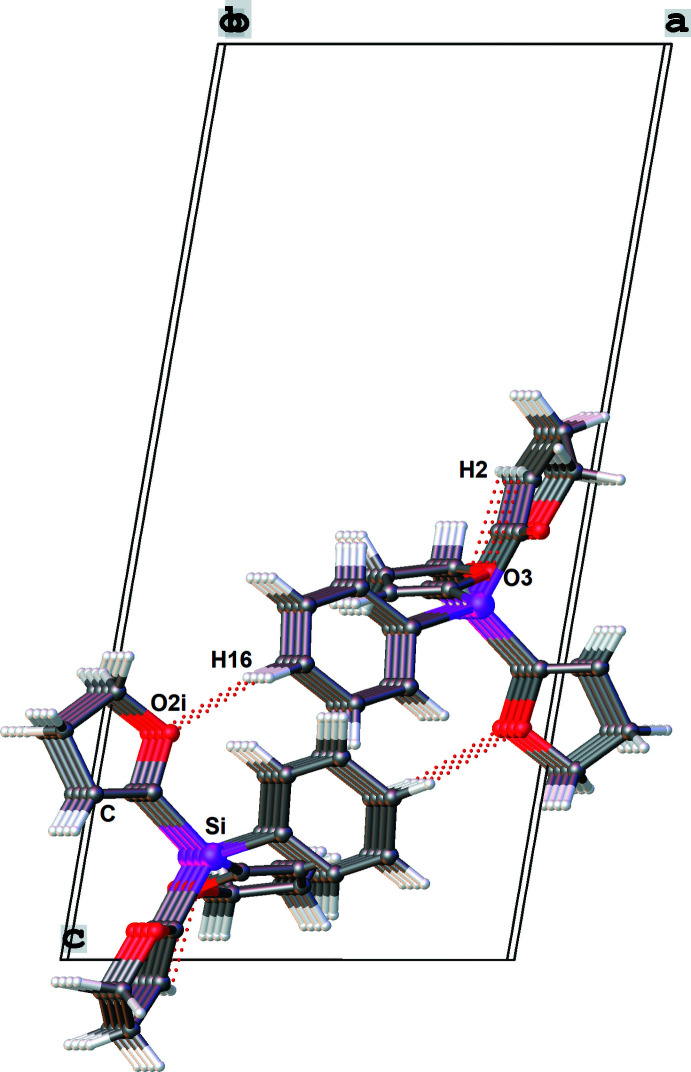
The crystal packing of compound **2** in a partial view along the *b* axis. C—H⋯O hydrogen bonds are shown as dotted lines. [Symmetry code: (i) −*x* + 1, *y* + 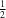, −*z* + 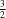].

**Figure 7:**
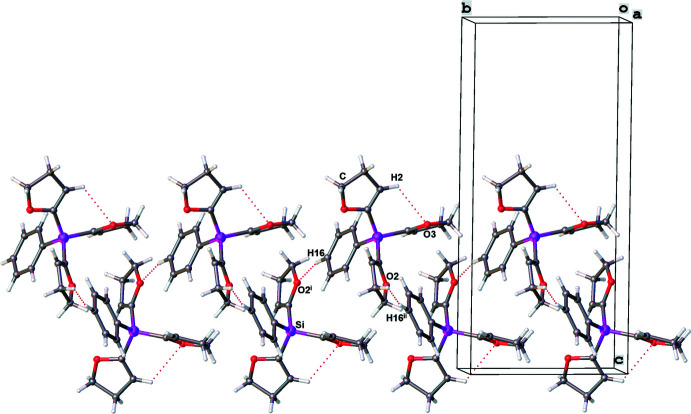
The crystal packing of compound **2** in a partial view along the *a* axis, showing inter­molecular and intra­molecular hydrogen bonds C16—H16⋯O2^i^ and C2—H2⋯O3. [Symmetry codes: (i) −*x* + 1, *y* + 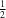, −*z* + 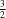; (ii) −*x* + 1, *y* − 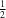, −*z* + 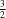].

**Figure 8:**
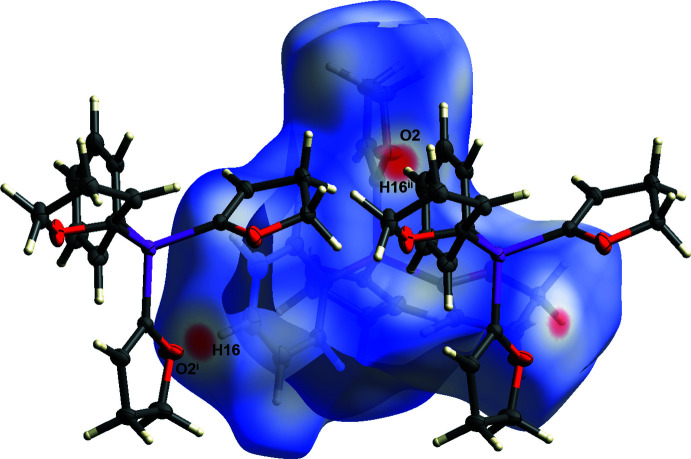
Hirshfeld surface analysis of **2** showing close contacts in the crystal. The weak hydrogen bond between oxygen atom O2 and the H16 hydrogen atom is labelled. [Symmetry codes: (i) −*x* + 1, *y* + 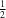, −*z* + 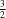; (ii) −*x* + 1, *y* − 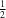, −*z* + 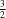].

**Figure 9:**
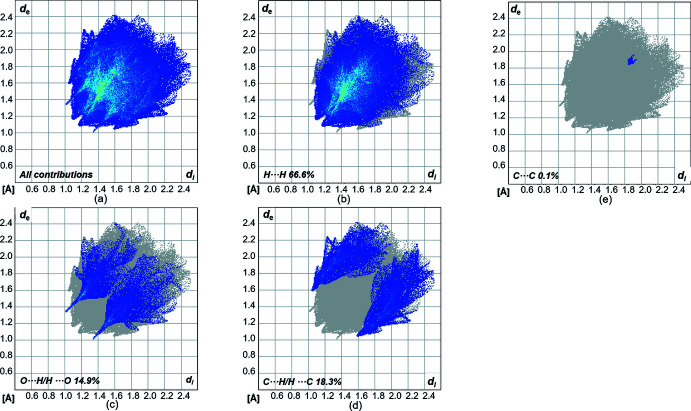
(*a*) Two-dimensional fingerprint plots of compound **2**, showing all contributions, and delineated (*b*)–(*e*) showing the contributions of atoms within specific inter­acting pairs (blue areas).

The crystal packing of compound **2** is illustrated in Fig. 6 ▸ and shows a ribbon-like supra­molecular network structure propagating along the *b-*axis direction. The mol­ecules are linked by a C—H⋯O hydrogen bond between the O2^i^ atom of a DHF group and the C16—H16_*para*_ group of the phenyl ring (Table 4 ▸), leading to the formation of chains with graph-set motif 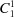
(8). Compared to compound **1** where the methyl group shows no hydrogen-bonding inter­actions, the phenyl group is important for the crystal packing, as emphasized in Fig. 7 ▸. Again, the strengths of the hydrogen bonds can be classified as weak (Desiraju & Steiner, 1999 ▸). A Hirshfeld surface analysis of **2** was carried out with *d*_norm_ in the range from −0.1662 to 1.2663 a.u.. The characteristic red spots in Fig. 8 ▸ indicate the C16—H16⋯O2^i^ inter­actions. The two-dimensional fingerprint plots are displayed in Fig. 9 ▸. Compared to compound **1**, the C⋯H/H⋯C contacts appear to be more important for **2** than the O⋯H/H⋯O contacts. Nevertheless, H⋯H, C⋯H/H⋯C and O⋯H/H⋯O are likewise significant contributors to the packing arrangement within the crystal structure.

## Synthesis and crystallization   

5-Li­thio-2,3-di­hydro­furan was prepared as described in the literature (Gevorgyan *et al.*, 1990 ▸). The subsequent implementation of the li­thia­ted species with the chloro­silane was also carried out as previously described (Erchak *et al.*, 1981 ▸; Gevorgyan *et al.*, 1997 ▸).

Tris(4,5-di­hydro­furan-2-yl)methyl­silane (**1**) is a colourless crystalline solid at room temperature:

^1^H NMR (400 MHz, C_6_H_6_): δ = 0.65 (*s*, 3H; SiC*H*_3_), 2.25 [*dt*, _3_ *J*_HH_ = 2.57 Hz, _3_ *J*_HH_ = 9.66 Hz, 6H; Si(CCHC*H*_2_)_3_], 4.06 [*t*, _3_ *J*_HH_ = 9.66 Hz, 6H; Si(COC*H*_2_)_3_], 5.59 [*t*, _3_ *J*_HH_ = 2.57 Hz, 3H; Si(CC*H*)_3_] ppm.

{^1^H}^13^C NMR (100 MHz, C_6_H_6_): δ = −5.7 (1C; Si*C*H_3_), 31.4 [3C; Si(CCH*C*H_2_)_3_], 70.9 [3C; Si(CO*C*H_2_)_3_], 115.6 [3C; Si(C*C*H)_3_], 157.5 [3C; Si(*C*O)_3_] ppm.

{^1^H}^29^Si NMR (79 MHz, C_6_H_6_): −36.65 [1Si; *Si*(DHF)_3_] ppm.

GC/EI–MS *t*_R_ = 5.40 min [353 K (1 min) – 40 K min^−1^ – 543 K (5.5 min)]; *m*/*z* (%): 250 (100) [*M* ^+^], 207 (4) [(*M* − C_2_H_3_O)^+^], 121 (56) [(DHFSiCCH]^+^], 97 (13) [(SiDHF)^+^].

Tris(4,5-di­hydro­furan-2-yl)phenyl­silane (**2**) is a colourless crystalline solid at room temperature:

^1^H NMR (400 MHz, C_6_H_6_): δ = 2.25 [*dt*, _3_ *J*_HH_ = 2.57 Hz, _3_ *J*_HH_ = 9.66 Hz, 6H; Si(CCHC*H*_2_)_3_], 4.07 [*t*, _3_ *J*_HH_ = 9.66 Hz, 6H; Si(COC*H*_2_)_3_], 5.72 [*t*, _3_ *J*_HH_ = 2.57 Hz, 3H; Si(CC*H*)_3_], 7.18–7.27 (*m*, 3H; Ph–*H*_ortho,para_), 8.11–8.14 (*m*, 2H; Ph–*H*_meta_) ppm.

{^1^H}^13^C NMR (100 MHz, C_6_H_6_): δ = 31.4 [3C; Si(CCH*C*H_2_)_3_], 71.1 [3C; Si(CO*C*H_2_)_3_]; 117.8 [3C; Si(C*C*H)_3_]; 128.4 (2C; Ph–*C*_ortho_); 130.8 (1C, Ph–*C*_para_); 134.3 (1C; Ph–*C*_ipso_); 136.3 (2C; Ph–*C*_meta_); 156.4 [3C; Si(*C*O)_3_] ppm.

{^1^H}^29^Si NMR (79 MHz, C_6_H_6_): −41.74 [1Si; *Si*(DHF)_3_] ppm.

GC/EI–MS *t*_R_ = 6.88 min [353 K (1 min) – 40 K min^−1^ – 543 K(5.5 min)]; m/z (%): 312 (100) [*M* ^+^], 255 (21) [(*M* − C_3_H_5_O)^+^], 105 (53) [(SiPh]^+^], 77 (12) [Ph^+^], 69 (6) [DHF^+^].

## Refinement   

Crystal data, data collection and structure refinement details are summarized in Table 6 ▸. For the IAM approach using *SHELXL* (Sheldrick, 2015*b* ▸), the H atoms were positioned geometrically (C—H = 0.95–1.00 Å) and were refined using a riding model, with *U*_iso_(H) = 1.2*U*_eq_(C) for CH_2_ and CH hydrogen atoms and *U*_iso_(H) = 1.5*U*_eq_(C) for CH_3_ hydrogen atoms. Hydrogen atoms H6, H8*A*,*B* and H11*A*,*B* for compound **1** and H2 and H16 for compound **2** were refined independently.

HARs were performed with the HARt implementation in *OLEX2* (Dolomanov *et al.*, 2009 ▸), using the restricted Khom–Sham method with the basis set x2c-TZVP. The results of previous IAM refinements using - served as an input (Fugel *et al.*, 2018 ▸). For the HAR approach, all H atoms were refined anistropically and independently.

## Supplementary Material

Crystal structure: contains datablock(s) 1, 1HAR, 2, 2HAR. DOI: 10.1107/S2056989020011470/wm5561sup1.cif

Structure factors: contains datablock(s) 1. DOI: 10.1107/S2056989020011470/wm55611sup2.hkl

Structure factors: contains datablock(s) 1HAR. DOI: 10.1107/S2056989020011470/wm55611HARsup4.hkl

Structure factors: contains datablock(s) 2. DOI: 10.1107/S2056989020011470/wm55612sup3.hkl

Structure factors: contains datablock(s) 2HAR. DOI: 10.1107/S2056989020011470/wm55612HARsup5.hkl

CCDC references: 2024677, 2024676, 2024675, 2024674

Additional supporting information:  crystallographic information; 3D view; checkCIF report

## Figures and Tables

**Table 1:** Selected geometric parameters of compound **1** (Å, °)

	IAM	HAR		IAM	HAR
Si1—C1	1.8664 (8)	1.8663 (5)	C1—Si1—C5	111.25 (4)	111.33 (2)
Si1—C5	1.8640 (8)	1.8643 (5)	C1—Si1—C9	106.48 (4)	106.55 (2)
Si1—C9	1.8610 (8)	1.8628 (5)	C1—Si1—C13	109.38 (4)	109.36 (2)
Si1—C13	1.8559 (9)	1.8570 (5)	C5—Si1—C9	107.10 (4)	107.14 (2)
			C5—Si1—C13	110.92 (4)	110.86 (2)
C1—C2	1.3312 (11)	1.3356 (6)	C9—Si1—C13	111.61 (4)	111.52 (2)
C5—C6	1.3315 (12)	1.3357 (6)			
C9—C10	1.3273 (12)	1.3294 (7)			

**Table 2:** Selected geometric parameters of compound **2** (Å, °)

	IAM	HAR		IAM	HAR
Si1—C1	1.8633 (9)	1.8643 (5)	C1—Si1—C5	107.26 (4)	107.29 (2)
Si1—C5	1.8638 (9)	1.8646 (5)	C1—Si1—C9	107.99 (4)	108.03 (2)
Si1—C9	1.8670 (9)	1.8680 (5)	C1—Si1—C13	112.97 (4)	112.96 (2)
Si1—C13	1.8662 (9)	1.8672 (5)	C5—Si1—C9	112.15 (4)	112.08 (2)
			C5—Si1—C13	109.53 (4)	109.47 (2)
C1—C2	1.3314 (12)	1.3350 (7)	C9—Si1—C13	107.01 (4)	107.08 (2)
C5—C6	1.3317 (12)	1.3348 (7)			
C9—C10	1.3348 (12)	1.3356 (7)			

**Table 3:** Hydrogen-bond geometry (Å, °) for **1** [Chem scheme1]

*D*—H⋯*A*	*D*—H	H⋯*A*	*D*⋯*A*	*D*—H⋯*A*
C6—H6⋯O1^i^	0.912 (15)	2.658 (15)	3.4264 (12)	142.5 (12)
C8—H8*A*⋯O3^ii^	1.005 (16)	2.587 (15)	3.3291 (13)	130.5 (11)
C11—H11*A*⋯O2^i^	0.944 (19)	2.538 (19)	3.4369 (14)	159.2 (15)

Symmetry codes: (i) 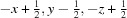; (ii) 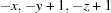.

**Table 4:** Hydrogen-bond geometry (Å, °) for **2** [Chem scheme1]

*D*—H⋯*A*	*D*—H	H⋯*A*	*D*⋯*A*	*D*—H⋯*A*
C16—H16⋯O2^i^	0.987 (18)	2.474 (18)	3.4394 (13)	165.9 (15)
C2—H2⋯O3	0.995 (17)	2.809 (17)	3.4238 (13)	120.6 (12)

Symmetry code: (i) 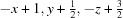.

**Table 5:** C—H bond length (Å) of the methine groups for IAM and HAR for compounds **1** and **2**

	**1**			**2**		
	C2—H2	C6—H6	C10—H10	C2—H2	C6—H6	C10—H10
IAM	0.9500	0.912 (15)^*a*^	0.9500	0.995 (17)^*a*^	0.9500	0.9500
HAR	1.084 (6)	1.070 (6)	1.088 (7)	1.079 (7)	1.077 (7)	1.049 (8)

Note: (*a*) Hydrogen atoms were refined independently.

**Table 6:** Experimental details

	**1** (IAM)	**1** (HAR)	**2** (IAM)	**2** (HAR)
Crystal data				
Chemical formula	C_13_H_18_O_3_Si	C_13_H_18_O_3_Si	C_18_H_20_O_3_Si	C_18_H_20_O_3_Si
*M*_r_	250.36	250.37	312.43	312.44
Crystal system, space group	Monoclinic, *P*2_1_/*n*	Monoclinic, *P*2_1_/*n*	Monoclinic, *P*2_1_/*c*	Monoclinic, *P*2_1_/*c*
Temperature (K)	100	100	100	100
*a*, *b*, *c* (Å)	7.9801 (4), 12.2381 (5), 13.3712 (7)	7.9801 (4), 12.2381 (5), 13.3712 (7)	9.4936 (6), 8.6802 (7), 19.747 (2)	9.4936 (6), 8.6802 (7), 19.747 (2)
β (°)	90.134 (2)	90.134 (2)	99.743 (4)	99.743 (4)
*V* (Å^3^)	1305.84 (11)	1305.84 (11)	1603.8 (2)	1603.8 (2)
*Z*	4	4	4	4
Radiation type	Mo *K*α	Mo *K*α	Mo *K*α	Mo *K*α
μ (mm^−1^)	0.17	0.17	0.16	0.16
Crystal size (mm)	0.39 × 0.14 × 0.07	0.39 × 0.14 × 0.07	1 × 0.58 × 0.36	1 × 0.58 × 0.36

Data collection				
Diffractometer	Bruker D8 Venture	Bruker D8 Venture	Bruker D8 Venture	Bruker D8 Venture
Absorption correction	Multi-scan (*SADABS*; Bruker, 2016 ▸)	Multi-scan (*SADABS*; Bruker, 2016 ▸)	Multi-scan (*SADABS*; Bruker, 2016 ▸)	Multi-scan (*SADABS*; Bruker, 2016 ▸)
*T*_min_, *T*_max_	0.536, 0.567	0.536, 0.567	0.484, 0.566	0.484, 0.566
No. of measured, independent and observed reflections	51391, 5737, 4936 [*I* > 2σ(*I*)]	51391, 4984, 4984 [*F* > 0 & *F*/σ(*F*) > 3.0 & |*F*_calc_| > 10^−3^]	25027, 5830, 5318 [*I* > 2σ(*I*)]	25027, 5359, 5359 [*F* > 0 & *F*/σ(*F*) > 3.0 & |*F*_calc_| > 10^−3^]
*R*_int_	0.034	0.034	0.030	0.030
(sin θ/λ)_max_ (Å^−1^)	0.807	0.807	0.758	0.758

Refinement				
*R*[*F* ^2^ > 2σ(*F* ^2^)], *wR*(*F* ^2^), *S*	0.035, 0.101, 1.06	0.023, 0.017, 1.94	0.037, 0.105, 1.06	0.024, 0.021, 2.07
No. of reflections	5737	5737	5830	5830
No. of parameters	175	316	207	379
H-atom treatment	H atoms treated by a mixture of independent and constrained refinement	All H-atom parameters refined	H atoms treated by a mixture of independent and constrained refinement	All H-atom parameters refined
Δρ_max_, Δρ_min_ (e Å^−3^)	0.55, −0.21	0.21, −0.22	0.47, −0.23	0.26, −0.18

Computer programs: *APEX2* (Bruker, 2018 ▸), *SAINT* (Bruker, 2016 ▸), *SHELXT* (Sheldrick, 2015*a* ▸), *SHELXL* (Sheldrick, 2015*b* ▸), *TONTO* (Jayatilaka & Grimwood, 2003 ▸), *OLEX2* (Dolomanov *et al.*, 2009 ▸), *Mercury* (Macrae *et al.*, 2020 ▸) and *publCIF* (Westrip, 2010 ▸).

## supplementary crystallographic information

### Tris(4,5-dihydrofuran-2-yl)methylsilane (1) Crystal data

**Table:**

C_13_H_18_O_3_Si	*F*(000) = 536
*M**_r_* = 250.36	*D*_x_ = 1.273 Mg m^−^^3^
Monoclinic, *P*2_1_/*n*	Mo *K*α radiation, λ = 0.71073 Å
*a* = 7.9801 (4) Å	Cell parameters from 9906 reflections
*b* = 12.2381 (5) Å	θ = 2.3–36.3°
*c* = 13.3712 (7) Å	µ = 0.17 mm^−^^1^
β = 90.134 (2)°	*T* = 100 K
*V* = 1305.84 (11) Å^3^	Block, colourless
*Z* = 4	0.39 × 0.14 × 0.07 mm

### Tris(4,5-dihydrofuran-2-yl)methylsilane (1) Data collection

**Table:**

Bruker D8 Venture diffractometer	5737 independent reflections
Radiation source: microfocus sealed X-ray tube, Incoatec Iµs	4936 reflections with *I* > 2σ(*I*)
HELIOS mirror optics monochromator	*R*_int_ = 0.034
Detector resolution: 10.4167 pixels mm^-1^	θ_max_ = 35.0°, θ_min_ = 2.3°
ω and φ scans	*h* = −12→12
Absorption correction: multi-scan (SADABS; Bruker, 2016)	*k* = −19→19
*T*_min_ = 0.536, *T*_max_ = 0.567	*l* = −21→21
51391 measured reflections	

### Tris(4,5-dihydrofuran-2-yl)methylsilane (1) Refinement

**Table:**

Refinement on *F*^2^	Primary atom site location: iterative
Least-squares matrix: full	Secondary atom site location: difference Fourier map
*R*[*F*^2^ > 2σ(*F*^2^)] = 0.035	Hydrogen site location: mixed
*wR*(*F*^2^) = 0.101	H atoms treated by a mixture of independent and constrained refinement
*S* = 1.06	*w* = 1/[σ^2^(*F*_o_^2^) + (0.048*P*)^2^ + 0.3601*P*] where *P* = (*F*_o_^2^ + 2*F*_c_^2^)/3
5737 reflections	(Δ/σ)_max_ = 0.001
175 parameters	Δρ_max_ = 0.55 e Å^−^^3^
0 restraints	Δρ_min_ = −0.21 e Å^−^^3^

### Tris(4,5-dihydrofuran-2-yl)methylsilane (1) Special details

**Table:**

Geometry. All esds (except the esd in the dihedral angle between two l.s. planes) are estimated using the full covariance matrix. The cell esds are taken into account individually in the estimation of esds in distances, angles and torsion angles; correlations between esds in cell parameters are only used when they are defined by crystal symmetry. An approximate (isotropic) treatment of cell esds is used for estimating esds involving l.s. planes.

### Tris(4,5-dihydrofuran-2-yl)methylsilane (1) Fractional atomic coordinates and isotropic or equivalent isotropic displacement parameters (Å^2^)

**Table:**

	*x*	*y*	*z*	*U*_iso_*/*U*_eq_	
Si1	0.18345 (3)	0.59411 (2)	0.25638 (2)	0.01471 (6)	
O1	0.35857 (8)	0.66915 (6)	0.09264 (5)	0.02745 (14)	
O2	0.27937 (10)	0.62127 (6)	0.45636 (5)	0.02791 (14)	
O3	−0.09090 (9)	0.45569 (7)	0.22765 (6)	0.02990 (15)	
C5	0.23012 (9)	0.54481 (6)	0.38536 (6)	0.01714 (13)	
C9	0.06571 (10)	0.48340 (6)	0.19167 (6)	0.01715 (13)	
C1	0.37989 (10)	0.61563 (7)	0.18350 (6)	0.01739 (13)	
C6	0.21595 (12)	0.44438 (7)	0.42270 (7)	0.02353 (16)	
C2	0.53927 (11)	0.59006 (9)	0.20150 (6)	0.02569 (18)	
H2	0.578431	0.552875	0.259375	0.031*	
C4	0.51776 (11)	0.66560 (9)	0.04067 (7)	0.02635 (17)	
H4A	0.546606	0.738778	0.014252	0.032*	
H4B	0.512329	0.613546	−0.015913	0.032*	
C13	0.06191 (11)	0.72335 (7)	0.25968 (7)	0.02361 (16)	
H13A	−0.045763	0.710554	0.292773	0.035*	
H13B	0.042121	0.748958	0.191220	0.035*	
H13C	0.125193	0.778791	0.296792	0.035*	
C10	0.11013 (13)	0.42563 (8)	0.11202 (8)	0.02825 (18)	
H10	0.213876	0.432901	0.077964	0.034*	
C7	0.26064 (14)	0.44446 (9)	0.53240 (7)	0.02880 (19)	
H7A	0.365266	0.403255	0.545241	0.035*	
H7B	0.169216	0.413336	0.573425	0.035*	
C11	−0.02632 (16)	0.34719 (9)	0.08321 (10)	0.0370 (2)	
C3	0.64834 (11)	0.62855 (10)	0.11701 (7)	0.0310 (2)	
H3A	0.718425	0.568501	0.090423	0.037*	
H3B	0.721462	0.689775	0.138009	0.037*	
C8	0.28359 (15)	0.56597 (9)	0.55268 (7)	0.03038 (19)	
C12	−0.14684 (16)	0.36115 (9)	0.16999 (10)	0.0395 (3)	
H12A	−0.262120	0.373096	0.144753	0.047*	
H12B	−0.146562	0.294897	0.212480	0.047*	
H6	0.1832 (19)	0.3836 (12)	0.3884 (11)	0.035 (4)*	
H8A	0.1907 (19)	0.5968 (12)	0.5948 (11)	0.035 (4)*	
H11A	0.015 (2)	0.2753 (16)	0.0766 (12)	0.053 (5)*	
H8B	0.392 (2)	0.5853 (13)	0.5845 (12)	0.042 (4)*	
H11B	−0.076 (2)	0.3644 (14)	0.0192 (13)	0.047 (4)*	

### Tris(4,5-dihydrofuran-2-yl)methylsilane (1) Atomic displacement parameters (Å^2^)

**Table:**

	*U*^11^	*U*^22^	*U*^33^	*U*^12^	*U*^13^	*U*^23^
Si1	0.01370 (9)	0.01620 (10)	0.01424 (9)	0.00092 (6)	0.00108 (7)	−0.00036 (6)
O1	0.0200 (3)	0.0374 (4)	0.0249 (3)	0.0039 (2)	0.0039 (2)	0.0152 (3)
O2	0.0421 (4)	0.0257 (3)	0.0159 (3)	−0.0068 (3)	−0.0033 (3)	−0.0018 (2)
O3	0.0213 (3)	0.0398 (4)	0.0286 (3)	−0.0118 (3)	0.0024 (2)	−0.0029 (3)
C5	0.0158 (3)	0.0207 (3)	0.0149 (3)	0.0010 (2)	0.0013 (2)	−0.0010 (2)
C9	0.0165 (3)	0.0180 (3)	0.0169 (3)	−0.0002 (2)	−0.0007 (2)	0.0012 (2)
C1	0.0163 (3)	0.0204 (3)	0.0154 (3)	−0.0006 (2)	0.0012 (2)	0.0017 (2)
C6	0.0299 (4)	0.0217 (4)	0.0190 (3)	0.0017 (3)	0.0001 (3)	0.0010 (3)
C2	0.0161 (3)	0.0447 (5)	0.0162 (3)	0.0010 (3)	0.0006 (3)	0.0068 (3)
C4	0.0208 (4)	0.0371 (5)	0.0211 (4)	−0.0031 (3)	0.0030 (3)	0.0080 (3)
C13	0.0232 (4)	0.0205 (3)	0.0271 (4)	0.0054 (3)	0.0017 (3)	−0.0009 (3)
C10	0.0278 (4)	0.0302 (4)	0.0267 (4)	0.0011 (3)	−0.0006 (3)	−0.0112 (3)
C7	0.0354 (5)	0.0320 (4)	0.0190 (4)	0.0062 (4)	0.0005 (3)	0.0060 (3)
C11	0.0436 (6)	0.0244 (4)	0.0428 (6)	0.0007 (4)	−0.0175 (5)	−0.0110 (4)
C3	0.0160 (3)	0.0553 (6)	0.0218 (4)	−0.0025 (4)	0.0017 (3)	0.0088 (4)
C8	0.0380 (5)	0.0382 (5)	0.0149 (3)	−0.0043 (4)	−0.0022 (3)	0.0001 (3)
C12	0.0399 (6)	0.0301 (5)	0.0486 (7)	−0.0174 (4)	−0.0110 (5)	0.0057 (4)

### Tris(4,5-dihydrofuran-2-yl)methylsilane (1) Geometric parameters (Å, º)

**Table:**

Si1—C5	1.8640 (8)	C4—C3	1.5259 (13)
Si1—C9	1.8610 (8)	C13—H13A	0.9800
Si1—C1	1.8664 (8)	C13—H13B	0.9800
Si1—C13	1.8559 (9)	C13—H13C	0.9800
O1—C1	1.3904 (10)	C10—H10	0.9500
O1—C4	1.4500 (11)	C10—C11	1.5011 (15)
O2—C5	1.3892 (10)	C7—H7A	0.9900
O2—C8	1.4552 (12)	C7—H7B	0.9900
O3—C9	1.3825 (10)	C7—C8	1.5226 (16)
O3—C12	1.4596 (13)	C11—C12	1.519 (2)
C5—C6	1.3315 (12)	C11—H11A	0.944 (19)
C9—C10	1.3273 (12)	C11—H11B	0.966 (18)
C1—C2	1.3312 (11)	C3—H3A	0.9900
C6—C7	1.5088 (13)	C3—H3B	0.9900
C6—H6	0.912 (15)	C8—H8A	1.005 (16)
C2—H2	0.9500	C8—H8B	0.995 (17)
C2—C3	1.5034 (13)	C12—H12A	0.9900
C4—H4A	0.9900	C12—H12B	0.9900
C4—H4B	0.9900		
			
C5—Si1—C1	111.25 (4)	H13B—C13—H13C	109.5
C9—Si1—C5	107.10 (4)	C9—C10—H10	124.7
C9—Si1—C1	106.48 (4)	C9—C10—C11	110.59 (9)
C13—Si1—C5	110.92 (4)	C11—C10—H10	124.7
C13—Si1—C9	111.61 (4)	C6—C7—H7A	111.4
C13—Si1—C1	109.38 (4)	C6—C7—H7B	111.4
C1—O1—C4	107.40 (6)	C6—C7—C8	101.64 (7)
C5—O2—C8	107.31 (7)	H7A—C7—H7B	109.3
C9—O3—C12	106.64 (8)	C8—C7—H7A	111.4
O2—C5—Si1	118.03 (6)	C8—C7—H7B	111.4
C6—C5—Si1	128.96 (6)	C10—C11—C12	101.09 (8)
C6—C5—O2	112.93 (7)	C10—C11—H11A	111.5 (11)
O3—C9—Si1	118.21 (6)	C10—C11—H11B	112.6 (10)
C10—C9—Si1	128.71 (7)	C12—C11—H11A	113.5 (10)
C10—C9—O3	113.07 (8)	C12—C11—H11B	113.2 (10)
O1—C1—Si1	114.93 (6)	H11A—C11—H11B	105.3 (14)
C2—C1—Si1	132.51 (6)	C2—C3—C4	101.55 (7)
C2—C1—O1	112.56 (7)	C2—C3—H3A	111.5
C5—C6—C7	110.11 (8)	C2—C3—H3B	111.5
C5—C6—H6	126.0 (10)	C4—C3—H3A	111.5
C7—C6—H6	123.9 (10)	C4—C3—H3B	111.5
C1—C2—H2	124.9	H3A—C3—H3B	109.3
C1—C2—C3	110.19 (8)	O2—C8—C7	107.10 (7)
C3—C2—H2	124.9	O2—C8—H8A	107.8 (9)
O1—C4—H4A	110.4	O2—C8—H8B	106.7 (9)
O1—C4—H4B	110.4	C7—C8—H8A	112.2 (8)
O1—C4—C3	106.62 (7)	C7—C8—H8B	114.4 (9)
H4A—C4—H4B	108.6	H8A—C8—H8B	108.4 (13)
C3—C4—H4A	110.4	O3—C12—C11	107.41 (8)
C3—C4—H4B	110.4	O3—C12—H12A	110.2
Si1—C13—H13A	109.5	O3—C12—H12B	110.2
Si1—C13—H13B	109.5	C11—C12—H12A	110.2
Si1—C13—H13C	109.5	C11—C12—H12B	110.2
H13A—C13—H13B	109.5	H12A—C12—H12B	108.5
H13A—C13—H13C	109.5		
			
Si1—C5—C6—C7	−177.42 (7)	C1—Si1—C5—C6	−103.58 (9)
Si1—C9—C10—C11	−178.07 (7)	C1—Si1—C9—O3	−176.31 (6)
Si1—C1—C2—C3	−179.13 (8)	C1—Si1—C9—C10	2.60 (9)
O1—C1—C2—C3	0.50 (12)	C1—O1—C4—C3	−12.68 (11)
O1—C4—C3—C2	12.29 (11)	C1—C2—C3—C4	−8.04 (12)
O2—C5—C6—C7	−0.69 (11)	C6—C7—C8—O2	−9.06 (11)
O3—C9—C10—C11	0.88 (12)	C4—O1—C1—Si1	−172.41 (6)
C5—Si1—C9—O3	64.58 (7)	C4—O1—C1—C2	7.89 (11)
C5—Si1—C9—C10	−116.51 (9)	C13—Si1—C5—O2	−42.17 (7)
C5—Si1—C1—O1	−169.89 (6)	C13—Si1—C5—C6	134.42 (8)
C5—Si1—C1—C2	9.73 (11)	C13—Si1—C9—O3	−57.01 (7)
C5—O2—C8—C7	9.17 (11)	C13—Si1—C9—C10	121.90 (9)
C5—C6—C7—C8	6.12 (11)	C13—Si1—C1—O1	−47.00 (7)
C9—Si1—C5—O2	−164.18 (6)	C13—Si1—C1—C2	132.62 (10)
C9—Si1—C5—C6	12.41 (9)	C10—C11—C12—O3	10.49 (12)
C9—Si1—C1—O1	73.74 (7)	C8—O2—C5—Si1	171.65 (7)
C9—Si1—C1—C2	−106.64 (10)	C8—O2—C5—C6	−5.47 (11)
C9—O3—C12—C11	−10.57 (11)	C12—O3—C9—Si1	−174.71 (7)
C9—C10—C11—C12	−7.14 (12)	C12—O3—C9—C10	6.21 (11)
C1—Si1—C5—O2	79.83 (7)		

### Tris(4,5-dihydrofuran-2-yl)methylsilane (1) Hydrogen-bond geometry (Å, º)

**Table:**

*D*—H···*A*	*D*—H	H···*A*	*D*···*A*	*D*—H···*A*
C6—H6···O1^i^	0.912 (15)	2.658 (15)	3.4264 (12)	142.5 (12)
C8—H8*A*···O3^ii^	1.005 (16)	2.587 (15)	3.3291 (13)	130.5 (11)
C11—H11*A*···O2^i^	0.944 (19)	2.538 (19)	3.4369 (14)	159.2 (15)

Symmetry codes: (i) −*x*+1/2, *y*−1/2, −*z*+1/2; (ii) −*x*, −*y*+1, −*z*+1.

### (1HAR) Crystal data

**Table:**

C_13_H_18_O_3_Si	*F*(000) = 536
*M**_r_* = 250.37	*D*_x_ = 1.273 Mg m^−^^3^
Monoclinic, *P*2_1_/*n*	Mo *K*α radiation, λ = 0.710730 Å
Hall symbol: -P 2yn	Cell parameters from 9906 reflections
*a* = 7.9801 (4) Å	θ = 2.3–36.3°
*b* = 12.2381 (5) Å	µ = 0.17 mm^−^^1^
*c* = 13.3712 (7) Å	*T* = 100 K
β = 90.134 (2)°	Block, colourless
*V* = 1305.84 (11) Å^3^	0.39 × 0.14 × 0.07 mm
*Z* = 4	

### (1HAR) Data collection

**Table:**

Bruker D8 Venture diffractometer	4984 reflections with *F* > 0 & *F*/σ(*F*) > 3.0 & |*F*_calc| > 10^−^^3^
Radiation source: microfocus sealed X-ray tube, Incoatec Iµs	*R*_int_ = 0.034
ω and φ scans	θ_max_ = 35.0°, θ_min_ = 2.3°
Absorption correction: multi-scan (SADABS; Bruker, 2016)	*h* = −12→12
*T*_min_ = 0.536, *T*_max_ = 0.567	*k* = −19→19
51391 measured reflections	*l* = −21→21
4984 independent reflections	

### (1HAR) Refinement

**Table:**

Refinement on *F*^2^	0 restraints
Least-squares matrix: full	0 constraints
*R*[*F*^2^ > 2σ(*F*^2^)] = 0.023	All H-atom parameters refined
*wR*(*F*^2^) = 0.017	Weighting scheme based on measured s.u.'s *w* = 1/σ(*F*)
*S* = 1.94	(Δ/σ)_max_ = 0.003
5737 reflections	Δρ_max_ = 0.21 e Å^−^^3^
316 parameters	Δρ_min_ = −0.21 e Å^−^^3^

### (1HAR) Special details

**Table:**

Refinement. HAR makes use of tailor-made aspherical atomic form factors calculated on-the-fly from a Hirshfeld-partitioned electron density (ED) - not from spherical-atom form factors.The ED is calculated from a gaussian basis set single determinant SCF wavefunction - either SCF or DFT - for a fragment of the crystal embedded in an electrostatic crystal field.If constraints were applied they are defined by zero eigenvalues of the least-squares hessian, see the value of _refine_ls_SVD_threshold.Specify symmetry and Friedel pair averaging.Only reflections which satisfy the threshold expression are listed below, and only they are considered observed, thus the *_gt, *_all and *_total data are always the same.

### (1HAR) Fractional atomic coordinates and isotropic or equivalent isotropic displacement parameters (Å^2^)

**Table:**

	*x*	*y*	*z*	*U*_iso_*/*U*_eq_	
Si1	0.183455 (16)	0.594139 (9)	0.256364 (10)	0.01424 (6)	
O1	0.35916 (4)	0.66896 (3)	0.09282 (3)	0.02767 (19)	
O2	0.27908 (5)	0.62086 (3)	0.45636 (3)	0.02813 (19)	
O3	−0.09045 (4)	0.45535 (3)	0.22732 (3)	0.0301 (2)	
C5	0.23019 (6)	0.54500 (3)	0.38543 (3)	0.0170 (2)	
C9	0.06532 (6)	0.48336 (3)	0.19172 (3)	0.0170 (2)	
C1	0.37970 (6)	0.61577 (3)	0.18334 (3)	0.0173 (2)	
C6	0.21614 (7)	0.44418 (4)	0.42274 (4)	0.0235 (3)	
C2	0.53945 (6)	0.58979 (4)	0.20154 (4)	0.0259 (3)	
H2	0.5844 (8)	0.5495 (6)	0.2686 (5)	0.061 (5)	
C4	0.51774 (7)	0.66579 (5)	0.04077 (4)	0.0268 (3)	
H4a	0.5037 (9)	0.6060 (6)	−0.0230 (5)	0.068 (6)	
H4b	0.5396 (8)	0.7450 (5)	0.0115 (6)	0.059 (5)	
C13	0.06171 (7)	0.72340 (4)	0.25969 (5)	0.0237 (3)	
H13a	−0.0569 (9)	0.7113 (6)	0.2949 (6)	0.059 (5)	
H13b	0.1302 (9)	0.7857 (5)	0.2996 (6)	0.057 (5)	
H13c	0.0403 (9)	0.7530 (5)	0.1851 (5)	0.053 (5)	
C10	0.11010 (7)	0.42565 (4)	0.11191 (4)	0.0284 (3)	
H10	0.2281 (9)	0.4377 (6)	0.0729 (6)	0.061 (5)	
C7	0.26051 (8)	0.44426 (5)	0.53234 (4)	0.0287 (3)	
H7a	0.1622 (10)	0.4119 (5)	0.5786 (6)	0.062 (6)	
H7b	0.3746 (10)	0.3987 (6)	0.5462 (6)	0.069 (6)	
C11	−0.02599 (8)	0.34719 (5)	0.08313 (5)	0.0365 (3)	
H11a	−0.0846 (11)	0.3677 (6)	0.0135 (6)	0.076 (6)	
C3	0.64824 (7)	0.62856 (6)	0.11700 (5)	0.0316 (3)	
H3a	0.7276 (10)	0.6974 (7)	0.1396 (6)	0.070 (6)	
H3b	0.7330 (9)	0.5667 (7)	0.0885 (6)	0.077 (6)	
C8	0.28357 (9)	0.56612 (5)	0.55253 (4)	0.0303 (3)	
H8a	0.4015 (11)	0.5861 (6)	0.5874 (6)	0.071 (6)	
C12	−0.14713 (9)	0.36145 (5)	0.17009 (6)	0.0397 (4)	
H12a	−0.1438 (12)	0.2899 (6)	0.2200 (7)	0.104 (8)	
H12b	−0.2711 (10)	0.3785 (6)	0.1467 (7)	0.077 (6)	
H6	0.1795 (9)	0.3737 (5)	0.3807 (5)	0.053 (5)	
H8b	0.1825 (11)	0.5983 (5)	0.5968 (5)	0.069 (6)	
H11b	0.0170 (10)	0.2642 (6)	0.0788 (7)	0.086 (7)	

### (1HAR) Atomic displacement parameters (Å^2^)

**Table:**

	*U*^11^	*U*^22^	*U*^33^	*U*^12^	*U*^13^	*U*^23^
Si1	0.01329 (6)	0.01572 (6)	0.01373 (6)	0.00096 (4)	0.00107 (4)	−0.00037 (5)
O1	0.02067 (18)	0.03649 (19)	0.0259 (2)	0.00403 (15)	0.00302 (15)	0.01491 (15)
O2	0.0418 (2)	0.02546 (17)	0.01715 (18)	−0.00624 (15)	−0.00304 (16)	−0.00187 (14)
O3	0.02182 (19)	0.0400 (2)	0.0283 (2)	−0.01124 (15)	0.00259 (16)	−0.00231 (16)
C5	0.0172 (2)	0.0196 (2)	0.0144 (2)	0.00110 (16)	0.00094 (17)	−0.00006 (17)
C9	0.0174 (2)	0.0178 (2)	0.0158 (2)	−0.00049 (16)	−0.00031 (17)	−0.00022 (16)
C1	0.0144 (2)	0.0215 (2)	0.0159 (2)	−0.00071 (16)	0.00120 (17)	0.00195 (16)
C6	0.0310 (3)	0.0206 (2)	0.0190 (3)	0.0011 (2)	0.0000 (2)	0.00156 (19)
C2	0.0153 (2)	0.0457 (3)	0.0166 (2)	0.0016 (2)	0.00047 (19)	0.0074 (2)
H2	0.028 (4)	0.121 (6)	0.032 (5)	0.013 (4)	−0.003 (4)	0.033 (5)
C4	0.0214 (3)	0.0372 (3)	0.0218 (3)	−0.0036 (2)	0.0024 (2)	0.0093 (2)
H4a	0.058 (5)	0.119 (7)	0.026 (5)	0.001 (5)	−0.007 (4)	−0.039 (5)
H4b	0.051 (5)	0.049 (4)	0.077 (6)	−0.015 (4)	0.010 (4)	0.019 (4)
C13	0.0232 (3)	0.0205 (2)	0.0275 (3)	0.0061 (2)	0.0015 (2)	−0.0010 (2)
H13a	0.040 (5)	0.065 (5)	0.073 (7)	0.010 (4)	0.024 (4)	0.006 (4)
H13b	0.064 (5)	0.033 (4)	0.074 (6)	0.006 (4)	−0.019 (5)	−0.026 (4)
H13c	0.069 (6)	0.044 (4)	0.045 (5)	0.017 (4)	−0.006 (4)	0.020 (4)
C10	0.0281 (3)	0.0307 (3)	0.0266 (3)	0.0009 (2)	0.0001 (2)	−0.0123 (2)
H10	0.046 (5)	0.079 (5)	0.058 (6)	−0.017 (4)	0.027 (4)	−0.036 (4)
C7	0.0354 (3)	0.0320 (3)	0.0188 (3)	0.0064 (2)	0.0007 (2)	0.0065 (2)
H7a	0.084 (6)	0.056 (5)	0.046 (6)	−0.018 (4)	0.015 (5)	0.013 (4)
H7b	0.067 (6)	0.077 (6)	0.062 (6)	0.035 (5)	−0.014 (5)	0.017 (4)
C11	0.0432 (4)	0.0245 (3)	0.0418 (4)	0.0001 (2)	−0.0167 (3)	−0.0106 (3)
H11a	0.086 (6)	0.101 (6)	0.041 (6)	−0.017 (5)	−0.026 (5)	−0.005 (5)
C3	0.0155 (3)	0.0572 (4)	0.0220 (3)	−0.0028 (3)	0.0020 (2)	0.0090 (3)
H3a	0.069 (6)	0.099 (6)	0.042 (5)	−0.037 (5)	−0.006 (4)	0.005 (5)
H3b	0.034 (5)	0.123 (7)	0.073 (7)	0.042 (5)	0.021 (4)	0.044 (5)
C8	0.0374 (3)	0.0381 (3)	0.0153 (3)	−0.0043 (3)	−0.0021 (2)	−0.0008 (2)
H8a	0.081 (6)	0.087 (6)	0.047 (6)	−0.041 (5)	−0.031 (5)	0.003 (4)
C12	0.0379 (4)	0.0320 (3)	0.0492 (4)	−0.0167 (3)	−0.0099 (3)	0.0057 (3)
H12a	0.149 (9)	0.035 (5)	0.128 (9)	−0.037 (5)	0.009 (7)	0.040 (5)
H12b	0.053 (6)	0.083 (6)	0.096 (8)	0.002 (5)	−0.027 (5)	−0.032 (5)
H6	0.091 (6)	0.034 (4)	0.033 (5)	−0.010 (4)	−0.010 (4)	−0.004 (3)
H8b	0.110 (7)	0.063 (5)	0.033 (5)	0.011 (5)	0.039 (5)	−0.005 (4)
H11b	0.088 (7)	0.047 (5)	0.124 (9)	0.001 (5)	−0.023 (6)	−0.037 (5)

### (1HAR) Geometric parameters (Å, º)

**Table:**

Si1—C5	1.8643 (5)	C6—H6	1.070 (6)
Si1—C9	1.8628 (5)	C2—H2	1.084 (6)
Si1—C1	1.8663 (5)	C4—H4a	1.129 (6)
Si1—C13	1.8570 (5)	C4—H4b	1.060 (6)
O1—C1	1.3837 (6)	C13—H13a	1.068 (7)
O1—C4	1.4461 (6)	C13—H13b	1.078 (6)
O2—C5	1.3827 (5)	C13—H13c	1.074 (7)
O2—C8	1.4502 (7)	C10—H10	1.088 (7)
O3—C9	1.3756 (6)	C7—H7a	1.075 (7)
O3—C12	1.4522 (7)	C7—H7b	1.083 (7)
C5—C6	1.3357 (6)	C11—H11a	1.071 (8)
C9—C10	1.3294 (7)	C11—H11b	1.073 (7)
C1—C2	1.3356 (6)	C3—H3a	1.096 (7)
C6—C7	1.5069 (7)	C3—H3b	1.085 (8)
C2—C3	1.5038 (8)	C8—H8a	1.078 (7)
C4—C3	1.5251 (8)	C8—H8b	1.077 (7)
C10—C11	1.4991 (8)	C12—H12a	1.101 (7)
C7—C8	1.5266 (8)	C12—H12b	1.058 (8)
C11—C12	1.5239 (10)		
			
Si1—C5—O2	118.29 (3)	C10—C11—C12	101.03 (5)
Si1—C9—O3	118.53 (3)	C5—C6—H6	124.9 (4)
Si1—C1—O1	115.25 (3)	C9—C10—H10	123.2 (3)
Si1—C5—C6	128.82 (4)	C1—C2—H2	125.0 (4)
Si1—C9—C10	128.50 (4)	C6—C7—H7a	112.9 (4)
Si1—C1—C2	132.27 (4)	C6—C7—H7b	111.2 (4)
Si1—C13—H13a	110.9 (4)	C2—C3—H3a	111.7 (4)
Si1—C13—H13b	110.4 (4)	C2—C3—H3b	113.9 (4)
Si1—C13—H13c	110.3 (4)	C4—C3—H3a	110.3 (4)
O1—C1—C2	112.48 (4)	C4—C3—H3b	113.5 (4)
O1—C4—C3	106.46 (4)	C10—C11—H11a	112.8 (4)
O2—C5—C6	112.81 (4)	C10—C11—H11b	112.8 (4)
O2—C8—C7	106.96 (4)	C7—C6—H6	125.1 (4)
O3—C9—C10	112.97 (4)	C7—C8—H8a	113.8 (4)
O3—C12—C11	107.18 (5)	C7—C8—H8b	111.4 (4)
O1—C4—H4a	107.2 (4)	C11—C10—H10	126.2 (4)
O1—C4—H4b	107.3 (4)	C11—C12—H12a	110.9 (5)
O2—C8—H8a	107.4 (4)	C11—C12—H12b	113.0 (5)
O2—C8—H8b	107.5 (4)	C3—C2—H2	125.1 (4)
O3—C12—H12a	107.6 (5)	C3—C4—H4a	112.1 (4)
O3—C12—H12b	106.9 (4)	C3—C4—H4b	114.1 (4)
C5—Si1—C9	107.14 (2)	C8—C7—H7a	110.2 (4)
C5—Si1—C1	111.33 (2)	C8—C7—H7b	111.8 (4)
C5—Si1—C13	110.86 (2)	C12—C11—H11a	111.1 (5)
C9—Si1—C1	106.55 (2)	C12—C11—H11b	110.7 (5)
C9—Si1—C13	111.52 (2)	H4a—C4—H4b	109.3 (6)
C1—Si1—C13	109.36 (2)	H13a—C13—H13b	109.2 (5)
C5—O2—C8	107.72 (4)	H13a—C13—H13c	108.4 (5)
C9—O3—C12	107.19 (4)	H13b—C13—H13c	107.5 (5)
C1—O1—C4	107.84 (4)	H7a—C7—H7b	109.0 (6)
C5—C6—C7	110.06 (5)	H11a—C11—H11b	108.3 (6)
C9—C10—C11	110.51 (5)	H3a—C3—H3b	105.9 (6)
C1—C2—C3	109.90 (5)	H8a—C8—H8b	109.5 (6)
C6—C7—C8	101.57 (4)	H12a—C12—H12b	110.9 (7)
C2—C3—C4	101.66 (4)		

### Tris(4,5-dihydrofuran-2-yl)phenylsilane (2) Crystal data

**Table:**

C_18_H_20_O_3_Si	*F*(000) = 664
*M**_r_* = 312.43	*D*_x_ = 1.294 Mg m^−^^3^
Monoclinic, *P*2_1_/*c*	Mo *K*α radiation, λ = 0.71073 Å
*a* = 9.4936 (6) Å	Cell parameters from 9914 reflections
*b* = 8.6802 (7) Å	θ = 2.6–30.5°
*c* = 19.747 (2) Å	µ = 0.16 mm^−^^1^
β = 99.743 (4)°	*T* = 100 K
*V* = 1603.8 (2) Å^3^	Block, colourless
*Z* = 4	1 × 0.58 × 0.36 mm

### Tris(4,5-dihydrofuran-2-yl)phenylsilane (2) Data collection

**Table:**

Bruker D8 Venture diffractometer	5830 independent reflections
Radiation source: microfocus sealed X-ray tube, Incoatec Iµs	5318 reflections with *I* > 2σ(*I*)
HELIOS mirror optics monochromator	*R*_int_ = 0.030
Detector resolution: 10.4167 pixels mm^-1^	θ_max_ = 32.6°, θ_min_ = 2.2°
φ and ω scans	*h* = −14→14
Absorption correction: multi-scan (SADABS; Bruker, 2016)	*k* = −13→10
*T*_min_ = 0.484, *T*_max_ = 0.566	*l* = −29→29
25027 measured reflections	

### Tris(4,5-dihydrofuran-2-yl)phenylsilane (2) Refinement

**Table:**

Refinement on *F*^2^	Primary atom site location: iterative
Least-squares matrix: full	Secondary atom site location: difference Fourier map
*R*[*F*^2^ > 2σ(*F*^2^)] = 0.037	Hydrogen site location: mixed
*wR*(*F*^2^) = 0.105	H atoms treated by a mixture of independent and constrained refinement
*S* = 1.06	*w* = 1/[σ^2^(*F*_o_^2^) + (0.053*P*)^2^ + 0.5057*P*] where *P* = (*F*_o_^2^ + 2*F*_c_^2^)/3
5830 reflections	(Δ/σ)_max_ = 0.001
207 parameters	Δρ_max_ = 0.47 e Å^−^^3^
0 restraints	Δρ_min_ = −0.23 e Å^−^^3^

### Tris(4,5-dihydrofuran-2-yl)phenylsilane (2) Special details

**Table:**

Geometry. All esds (except the esd in the dihedral angle between two l.s. planes) are estimated using the full covariance matrix. The cell esds are taken into account individually in the estimation of esds in distances, angles and torsion angles; correlations between esds in cell parameters are only used when they are defined by crystal symmetry. An approximate (isotropic) treatment of cell esds is used for estimating esds involving l.s. planes.

### Tris(4,5-dihydrofuran-2-yl)phenylsilane (2) Fractional atomic coordinates and isotropic or equivalent isotropic displacement parameters (Å^2^)

**Table:**

	*x*	*y*	*z*	*U*_iso_*/*U*_eq_	
Si1	0.74752 (2)	0.58226 (3)	0.61267 (2)	0.01607 (7)	
O1	0.84911 (9)	0.80595 (8)	0.53080 (4)	0.02920 (16)	
O2	0.88956 (8)	0.53532 (11)	0.74733 (4)	0.03192 (18)	
H16	0.2962 (19)	0.928 (2)	0.6921 (9)	0.047 (5)*	
O3	0.76383 (8)	0.26869 (8)	0.57693 (4)	0.02727 (15)	
C1	0.80711 (9)	0.65298 (10)	0.53302 (4)	0.01795 (15)	
C2	0.81525 (11)	0.57638 (11)	0.47539 (5)	0.02286 (17)	
C3	0.86553 (11)	0.68235 (12)	0.42379 (5)	0.02608 (18)	
H3A	0.789037	0.701732	0.383993	0.031*	
H3B	0.950565	0.640314	0.407290	0.031*	
C4	0.90202 (13)	0.82803 (12)	0.46668 (6)	0.0301 (2)	
H4A	1.006696	0.844635	0.475727	0.036*	
H4B	0.856062	0.918995	0.441956	0.036*	
C5	0.90600 (9)	0.58711 (10)	0.68271 (4)	0.01867 (15)	
C6	1.03720 (10)	0.63899 (12)	0.68063 (5)	0.02363 (17)	
H6	1.068129	0.679122	0.640851	0.028*	
C7	1.12966 (10)	0.62499 (14)	0.75036 (5)	0.02814 (19)	
H7A	1.165349	0.726797	0.768255	0.034*	
H7B	1.211792	0.555409	0.749084	0.034*	
C8	1.02544 (11)	0.55684 (15)	0.79288 (5)	0.0315 (2)	
H8A	1.061678	0.456899	0.812821	0.038*	
H8B	1.013588	0.627441	0.830884	0.038*	
C9	0.67580 (9)	0.38311 (10)	0.59588 (4)	0.01827 (15)	
C10	0.54408 (10)	0.33313 (11)	0.59899 (5)	0.02234 (16)	
H10	0.469616	0.395021	0.611087	0.027*	
C11	0.53018 (12)	0.16494 (12)	0.58064 (6)	0.0311 (2)	
H11A	0.461907	0.148488	0.537473	0.037*	
H11B	0.499404	0.103890	0.617896	0.037*	
C12	0.68264 (12)	0.12463 (11)	0.57197 (6)	0.0299 (2)	
H12A	0.725657	0.052135	0.608368	0.036*	
H12B	0.682858	0.075547	0.526732	0.036*	
C13	0.60089 (9)	0.70098 (10)	0.63790 (4)	0.01831 (15)	
C14	0.46938 (10)	0.71555 (11)	0.59371 (5)	0.02264 (16)	
H14	0.457310	0.669609	0.549436	0.027*	
C15	0.35611 (11)	0.79615 (12)	0.61354 (6)	0.0288 (2)	
H15	0.267229	0.802831	0.583237	0.035*	
C16	0.37284 (12)	0.86642 (13)	0.67724 (7)	0.0321 (2)	
C17	0.50218 (14)	0.85581 (14)	0.72086 (6)	0.0345 (2)	
H17	0.514213	0.905500	0.764322	0.041*	
C18	0.61587 (12)	0.77307 (12)	0.70212 (5)	0.02617 (18)	
H18	0.703783	0.765721	0.733122	0.031*	
H2	0.7893 (18)	0.466 (2)	0.4669 (8)	0.039 (4)*	

### Tris(4,5-dihydrofuran-2-yl)phenylsilane (2) Atomic displacement parameters (Å^2^)

**Table:**

	*U*^11^	*U*^22^	*U*^33^	*U*^12^	*U*^13^	*U*^23^
Si1	0.01588 (11)	0.01536 (11)	0.01726 (11)	−0.00151 (7)	0.00365 (8)	−0.00197 (7)
O1	0.0477 (4)	0.0159 (3)	0.0283 (3)	−0.0049 (3)	0.0187 (3)	−0.0032 (2)
O2	0.0213 (3)	0.0488 (5)	0.0239 (3)	−0.0110 (3)	−0.0011 (2)	0.0123 (3)
O3	0.0240 (3)	0.0166 (3)	0.0415 (4)	0.0008 (2)	0.0065 (3)	−0.0049 (3)
C1	0.0172 (3)	0.0165 (3)	0.0208 (3)	−0.0008 (3)	0.0051 (3)	−0.0013 (3)
C2	0.0258 (4)	0.0220 (4)	0.0221 (4)	−0.0058 (3)	0.0079 (3)	−0.0042 (3)
C3	0.0281 (4)	0.0297 (5)	0.0223 (4)	−0.0047 (4)	0.0097 (3)	−0.0026 (3)
C4	0.0422 (6)	0.0218 (4)	0.0309 (5)	−0.0046 (4)	0.0196 (4)	−0.0009 (4)
C5	0.0185 (3)	0.0178 (3)	0.0195 (3)	−0.0018 (3)	0.0026 (3)	−0.0009 (3)
C6	0.0194 (4)	0.0282 (4)	0.0232 (4)	−0.0045 (3)	0.0035 (3)	0.0000 (3)
C7	0.0194 (4)	0.0338 (5)	0.0294 (4)	−0.0052 (4)	−0.0012 (3)	0.0032 (4)
C8	0.0250 (4)	0.0425 (6)	0.0247 (4)	−0.0097 (4)	−0.0024 (3)	0.0045 (4)
C9	0.0196 (3)	0.0161 (3)	0.0187 (3)	−0.0009 (3)	0.0021 (3)	−0.0013 (3)
C10	0.0212 (4)	0.0219 (4)	0.0239 (4)	−0.0048 (3)	0.0035 (3)	−0.0002 (3)
C11	0.0311 (5)	0.0215 (4)	0.0392 (5)	−0.0094 (4)	0.0014 (4)	0.0020 (4)
C12	0.0324 (5)	0.0154 (4)	0.0377 (5)	−0.0002 (3)	−0.0060 (4)	−0.0030 (4)
C13	0.0200 (3)	0.0166 (3)	0.0197 (3)	−0.0012 (3)	0.0072 (3)	−0.0011 (3)
C14	0.0208 (4)	0.0206 (4)	0.0269 (4)	0.0013 (3)	0.0052 (3)	−0.0026 (3)
C15	0.0210 (4)	0.0213 (4)	0.0458 (6)	0.0003 (3)	0.0109 (4)	0.0002 (4)
C16	0.0344 (5)	0.0243 (4)	0.0439 (6)	0.0036 (4)	0.0247 (5)	0.0019 (4)
C17	0.0489 (6)	0.0325 (5)	0.0265 (5)	0.0084 (5)	0.0188 (4)	−0.0030 (4)
C18	0.0323 (5)	0.0264 (4)	0.0204 (4)	0.0032 (4)	0.0061 (3)	−0.0042 (3)

### Tris(4,5-dihydrofuran-2-yl)phenylsilane (2) Geometric parameters (Å, º)

**Table:**

Si1—C1	1.8633 (9)	C7—C8	1.5215 (15)
Si1—C5	1.8638 (9)	C8—H8A	0.9900
Si1—C9	1.8670 (9)	C8—H8B	0.9900
Si1—C13	1.8662 (9)	C9—C10	1.3348 (12)
O1—C1	1.3892 (11)	C10—H10	0.9500
O1—C4	1.4518 (12)	C10—C11	1.5046 (14)
O2—C5	1.3865 (11)	C11—H11A	0.9900
O2—C8	1.4544 (12)	C11—H11B	0.9900
O3—C9	1.3894 (11)	C11—C12	1.5267 (17)
O3—C12	1.4635 (12)	C12—H12A	0.9900
C1—C2	1.3314 (12)	C12—H12B	0.9900
C2—C3	1.5090 (13)	C13—C14	1.4027 (13)
C2—H2	0.995 (17)	C13—C18	1.3995 (12)
C3—H3A	0.9900	C14—H14	0.9500
C3—H3B	0.9900	C14—C15	1.3934 (13)
C3—C4	1.5288 (15)	C15—H15	0.9500
C4—H4A	0.9900	C15—C16	1.3827 (17)
C4—H4B	0.9900	C16—H16	0.987 (18)
C5—C6	1.3317 (12)	C16—C17	1.3785 (19)
C6—H6	0.9500	C17—H17	0.9500
C6—C7	1.5074 (14)	C17—C18	1.3975 (15)
C7—H7A	0.9900	C18—H18	0.9500
C7—H7B	0.9900		
			
C1—Si1—C5	107.26 (4)	O2—C8—H8B	110.2
C1—Si1—C9	107.99 (4)	C7—C8—H8A	110.2
C1—Si1—C13	112.97 (4)	C7—C8—H8B	110.2
C5—Si1—C9	112.15 (4)	H8A—C8—H8B	108.5
C5—Si1—C13	109.53 (4)	O3—C9—Si1	119.46 (6)
C13—Si1—C9	107.01 (4)	C10—C9—Si1	127.52 (7)
C1—O1—C4	107.29 (7)	C10—C9—O3	113.01 (8)
C5—O2—C8	107.54 (7)	C9—C10—H10	124.8
C9—O3—C12	107.15 (8)	C9—C10—C11	110.42 (9)
O1—C1—Si1	118.11 (6)	C11—C10—H10	124.8
C2—C1—Si1	128.81 (7)	C10—C11—H11A	111.4
C2—C1—O1	113.08 (8)	C10—C11—H11B	111.4
C1—C2—C3	110.09 (8)	C10—C11—C12	101.72 (8)
C1—C2—H2	125.1 (10)	H11A—C11—H11B	109.3
C3—C2—H2	124.8 (9)	C12—C11—H11A	111.4
C2—C3—H3A	111.5	C12—C11—H11B	111.4
C2—C3—H3B	111.5	O3—C12—C11	107.17 (8)
C2—C3—C4	101.42 (8)	O3—C12—H12A	110.3
H3A—C3—H3B	109.3	O3—C12—H12B	110.3
C4—C3—H3A	111.5	C11—C12—H12A	110.3
C4—C3—H3B	111.5	C11—C12—H12B	110.3
O1—C4—C3	107.13 (8)	H12A—C12—H12B	108.5
O1—C4—H4A	110.3	C14—C13—Si1	120.61 (7)
O1—C4—H4B	110.3	C18—C13—Si1	121.52 (7)
C3—C4—H4A	110.3	C18—C13—C14	117.82 (8)
C3—C4—H4B	110.3	C13—C14—H14	119.4
H4A—C4—H4B	108.5	C15—C14—C13	121.22 (9)
O2—C5—Si1	118.24 (6)	C15—C14—H14	119.4
C6—C5—Si1	128.79 (7)	C14—C15—H15	119.9
C6—C5—O2	112.93 (8)	C16—C15—C14	120.12 (10)
C5—C6—H6	124.8	C16—C15—H15	119.9
C5—C6—C7	110.34 (8)	C15—C16—H16	122.2 (11)
C7—C6—H6	124.8	C17—C16—H16	118.3 (10)
C6—C7—H7A	111.4	C17—C16—C15	119.48 (9)
C6—C7—H7B	111.4	C16—C17—H17	119.5
C6—C7—C8	101.71 (8)	C16—C17—C18	121.00 (10)
H7A—C7—H7B	109.3	C18—C17—H17	119.5
C8—C7—H7A	111.4	C13—C18—H18	119.8
C8—C7—H7B	111.4	C17—C18—C13	120.34 (10)
O2—C8—C7	107.47 (8)	C17—C18—H18	119.8
O2—C8—H8A	110.2		
			
Si1—C1—C2—C3	178.37 (7)	C6—C7—C8—O2	0.87 (12)
Si1—C5—C6—C7	178.02 (7)	C8—O2—C5—Si1	−177.67 (7)
Si1—C9—C10—C11	179.13 (7)	C8—O2—C5—C6	0.44 (12)
Si1—C13—C14—C15	176.12 (8)	C9—Si1—C1—O1	172.60 (7)
Si1—C13—C18—C17	−177.23 (8)	C9—Si1—C1—C2	−7.02 (10)
O1—C1—C2—C3	−1.27 (12)	C9—Si1—C5—O2	−59.87 (8)
O2—C5—C6—C7	0.15 (12)	C9—Si1—C5—C6	122.36 (9)
O3—C9—C10—C11	−0.06 (11)	C9—Si1—C13—C14	−58.05 (8)
C1—Si1—C5—O2	−178.26 (7)	C9—Si1—C13—C18	119.39 (8)
C1—Si1—C5—C6	3.96 (10)	C9—O3—C12—C11	7.11 (11)
C1—Si1—C9—O3	59.03 (8)	C9—C10—C11—C12	4.34 (11)
C1—Si1—C9—C10	−120.11 (9)	C10—C11—C12—O3	−6.80 (11)
C1—Si1—C13—C14	60.67 (8)	C12—O3—C9—Si1	176.20 (6)
C1—Si1—C13—C18	−121.89 (8)	C12—O3—C9—C10	−4.54 (11)
C1—O1—C4—C3	9.46 (12)	C13—Si1—C1—O1	54.45 (8)
C1—C2—C3—C4	6.79 (11)	C13—Si1—C1—C2	−125.17 (9)
C2—C3—C4—O1	−9.64 (11)	C13—Si1—C5—O2	58.79 (8)
C4—O1—C1—Si1	175.01 (7)	C13—Si1—C5—C6	−118.98 (9)
C4—O1—C1—C2	−5.31 (12)	C13—Si1—C9—O3	−179.07 (7)
C5—Si1—C1—O1	−66.35 (8)	C13—Si1—C9—C10	1.79 (10)
C5—Si1—C1—C2	114.04 (9)	C13—C14—C15—C16	1.35 (15)
C5—Si1—C9—O3	−58.93 (8)	C14—C13—C18—C17	0.28 (15)
C5—Si1—C9—C10	121.92 (9)	C14—C15—C16—C17	−0.10 (16)
C5—Si1—C13—C14	−179.84 (7)	C15—C16—C17—C18	−1.03 (17)
C5—Si1—C13—C18	−2.40 (9)	C16—C17—C18—C13	0.94 (17)
C5—O2—C8—C7	−0.84 (13)	C18—C13—C14—C15	−1.42 (14)
C5—C6—C7—C8	−0.64 (12)		

### Tris(4,5-dihydrofuran-2-yl)phenylsilane (2) Hydrogen-bond geometry (Å, º)

**Table:**

*D*—H···*A*	*D*—H	H···*A*	*D*···*A*	*D*—H···*A*
C16—H16···O2^i^	0.987 (18)	2.474 (18)	3.4394 (13)	165.9 (15)
C2—H2···O3	0.995 (17)	2.809 (17)	3.4238 (13)	120.6 (12)

Symmetry code: (i) −*x*+1, *y*+1/2, −*z*+3/2.

### (2HAR) Crystal data

**Table:**

C_18_H_20_O_3_Si	*F*(000) = 664
*M**_r_* = 312.44	*D*_x_ = 1.294 Mg m^−^^3^
Monoclinic, *P*2_1_/*c*	Mo *K*α radiation, λ = 0.710730 Å
Hall symbol: -P 2ybc	Cell parameters from 9914 reflections
*a* = 9.4936 (6) Å	θ = 2.6–30.5°
*b* = 8.6802 (7) Å	µ = 0.16 mm^−^^1^
*c* = 19.747 (2) Å	*T* = 100 K
β = 99.743 (4)°	Block, colourless
*V* = 1603.8 (2) Å^3^	1 × 0.58 × 0.36 mm
*Z* = 4	

### (2HAR) Data collection

**Table:**

Bruker D8 Venture diffractometer	5359 reflections with *F* > 0 & *F*/σ(*F*) > 3.0 & |*F*_calc| > 10^−^^3^
Radiation source: microfocus sealed X-ray tube, Incoatec Iµs	*R*_int_ = 0.030
φ and ω scans	θ_max_ = 32.6°, θ_min_ = 2.2°
Absorption correction: multi-scan (SADABS; Bruker, 2016)	*h* = −14→14
*T*_min_ = 0.484, *T*_max_ = 0.566	*k* = −13→10
25027 measured reflections	*l* = −29→29
5359 independent reflections	

### (2HAR) Refinement

**Table:**

Refinement on *F*^2^	0 restraints
Least-squares matrix: full	0 constraints
*R*[*F*^2^ > 2σ(*F*^2^)] = 0.024	All H-atom parameters refined
*wR*(*F*^2^) = 0.021	Weighting scheme based on measured s.u.'s *w* = 1/σ(*F*)
*S* = 2.07	(Δ/σ)_max_ = 0.002
5830 reflections	Δρ_max_ = 0.26 e Å^−^^3^
379 parameters	Δρ_min_ = −0.17 e Å^−^^3^

### (2HAR) Special details

**Table:**

Refinement. HAR makes use of tailor-made aspherical atomic form factors calculated on-the-fly from a Hirshfeld-partitioned electron density (ED) - not from spherical-atom form factors.The ED is calculated from a gaussian basis set single determinant SCF wavefunction - either SCF or DFT - for a fragment of the crystal embedded in an electrostatic crystal field.If constraints were applied they are defined by zero eigenvalues of the least-squares hessian, see the value of _refine_ls_SVD_threshold.Specify symmetry and Friedel pair averaging.Only reflections which satisfy the threshold expression are listed below, and only they are considered observed, thus the *_gt, *_all and *_total data are always the same.

### (2HAR) Fractional atomic coordinates and isotropic or equivalent isotropic displacement parameters (Å^2^)

**Table:**

	*x*	*y*	*z*	*U*_iso_*/*U*_eq_	
Si1	0.747546 (14)	0.582292 (15)	0.612664 (7)	0.01528 (6)	
O1	0.84901 (5)	0.80558 (4)	0.53062 (2)	0.0292 (2)	
O2	0.89013 (4)	0.53540 (5)	0.74723 (2)	0.0314 (2)	
H16	0.2846 (9)	0.9319 (11)	0.6920 (5)	0.074 (7)	
O3	0.76344 (4)	0.26862 (4)	0.57700 (2)	0.0272 (2)	
C1	0.80726 (5)	0.65332 (6)	0.53303 (2)	0.0174 (2)	
C2	0.81512 (6)	0.57618 (7)	0.47531 (3)	0.0227 (2)	
C3	0.86534 (7)	0.68228 (7)	0.42380 (3)	0.0264 (3)	
H3a	0.9585 (9)	0.6409 (10)	0.4044 (5)	0.063 (6)	
H3b	0.7823 (9)	0.7044 (12)	0.3813 (4)	0.066 (6)	
C4	0.90164 (8)	0.82785 (7)	0.46665 (3)	0.0301 (3)	
H4a	0.8576 (12)	0.9288 (11)	0.4439 (5)	0.086 (8)	
H4b	1.0174 (10)	0.8428 (12)	0.4793 (5)	0.078 (7)	
C5	0.90593 (5)	0.58701 (6)	0.68281 (2)	0.0183 (2)	
C6	1.03741 (6)	0.63895 (7)	0.68061 (3)	0.0233 (2)	
H6	1.0707 (8)	0.6836 (11)	0.6349 (4)	0.054 (6)	
C7	1.12977 (6)	0.62511 (8)	0.75034 (3)	0.0281 (3)	
H7a	1.2215 (9)	0.5525 (13)	0.7488 (5)	0.076 (7)	
H7b	1.1704 (10)	0.7364 (10)	0.7693 (5)	0.071 (7)	
C8	1.02519 (7)	0.55702 (9)	0.79273 (3)	0.0318 (3)	
H8a	1.0062 (10)	0.6346 (16)	0.8331 (6)	0.101 (9)	
H8b	1.0587 (10)	0.4467 (14)	0.8157 (7)	0.101 (9)	
C9	0.67596 (5)	0.38297 (6)	0.59582 (2)	0.0176 (2)	
C10	0.54408 (6)	0.33323 (6)	0.59894 (3)	0.0221 (2)	
H10	0.4616 (8)	0.4016 (9)	0.6120 (5)	0.051 (6)	
C11	0.53018 (7)	0.16494 (7)	0.58067 (4)	0.0312 (3)	
H11a	0.4968 (10)	0.1008 (11)	0.6216 (5)	0.069 (7)	
H11b	0.4532 (8)	0.1460 (10)	0.5330 (5)	0.058 (6)	
C12	0.68277 (7)	0.12475 (7)	0.57208 (3)	0.0298 (3)	
H12a	0.6906 (10)	0.0754 (10)	0.5224 (5)	0.064 (6)	
H12b	0.7323 (10)	0.0499 (10)	0.6124 (5)	0.076 (7)	
C13	0.60087 (5)	0.70104 (6)	0.63796 (2)	0.0178 (2)	
C14	0.46947 (6)	0.71578 (6)	0.59372 (3)	0.0222 (2)	
H14	0.4539 (8)	0.6612 (10)	0.5433 (4)	0.050 (5)	
C15	0.35609 (6)	0.79613 (7)	0.61351 (3)	0.0287 (3)	
H15	0.2540 (8)	0.8044 (11)	0.5787 (5)	0.062 (6)	
C16	0.37264 (7)	0.86670 (7)	0.67727 (3)	0.0322 (3)	
C17	0.50259 (7)	0.85587 (7)	0.72103 (3)	0.0340 (3)	
H17	0.5195 (10)	0.9123 (11)	0.7706 (5)	0.072 (7)	
C18	0.61593 (7)	0.77294 (7)	0.70210 (3)	0.0260 (3)	
H2	0.7895 (10)	0.4556 (9)	0.4679 (4)	0.056 (6)	
H18	0.7151 (9)	0.7604 (10)	0.7373 (4)	0.055 (6)	

### (2HAR) Atomic displacement parameters (Å^2^)

**Table:**

	*U*^11^	*U*^22^	*U*^33^	*U*^12^	*U*^13^	*U*^23^
Si1	0.01511 (6)	0.01445 (6)	0.01659 (6)	−0.00151 (5)	0.00356 (5)	−0.00195 (5)
O1	0.0473 (2)	0.01601 (18)	0.0284 (2)	−0.00392 (16)	0.01778 (19)	−0.00359 (16)
O2	0.02147 (18)	0.0467 (3)	0.02463 (18)	−0.01067 (18)	0.00006 (15)	0.01162 (18)
H16	0.062 (5)	0.073 (7)	0.103 (9)	0.024 (5)	0.060 (6)	−0.008 (6)
O3	0.02383 (18)	0.01780 (18)	0.0400 (2)	0.00088 (14)	0.00591 (17)	−0.00434 (16)
C1	0.0182 (2)	0.0164 (2)	0.0186 (2)	−0.00123 (17)	0.00588 (18)	−0.00205 (18)
C2	0.0270 (2)	0.0215 (3)	0.0212 (2)	−0.0059 (2)	0.0085 (2)	−0.0053 (2)
C3	0.0286 (3)	0.0308 (3)	0.0217 (3)	−0.0047 (2)	0.0098 (2)	−0.0025 (2)
H3a	0.078 (6)	0.053 (6)	0.073 (8)	−0.009 (5)	0.058 (6)	−0.016 (5)
H3b	0.053 (5)	0.113 (9)	0.030 (5)	−0.002 (5)	0.002 (5)	0.014 (5)
C4	0.0428 (3)	0.0204 (3)	0.0317 (3)	−0.0047 (2)	0.0195 (3)	−0.0010 (2)
H4a	0.156 (10)	0.044 (6)	0.070 (8)	0.036 (6)	0.054 (8)	0.024 (6)
H4b	0.061 (6)	0.102 (9)	0.082 (9)	−0.049 (6)	0.048 (6)	−0.050 (7)
C5	0.0165 (2)	0.0183 (2)	0.0196 (2)	−0.00281 (18)	0.00220 (18)	−0.00111 (18)
C6	0.0181 (2)	0.0284 (3)	0.0235 (2)	−0.0054 (2)	0.0035 (2)	0.0002 (2)
H6	0.050 (5)	0.084 (7)	0.035 (5)	−0.011 (5)	0.024 (4)	0.011 (5)
C7	0.0190 (3)	0.0341 (3)	0.0293 (3)	−0.0051 (2)	−0.0015 (2)	0.0035 (2)
H7a	0.020 (4)	0.133 (10)	0.075 (7)	0.018 (5)	0.006 (5)	0.033 (7)
H7b	0.101 (8)	0.041 (6)	0.058 (6)	−0.050 (6)	−0.022 (6)	0.004 (5)
C8	0.0260 (3)	0.0430 (4)	0.0244 (3)	−0.0099 (3)	−0.0016 (2)	0.0054 (3)
H8a	0.052 (6)	0.175 (13)	0.079 (8)	−0.028 (7)	0.022 (6)	−0.088 (9)
H8b	0.055 (7)	0.102 (9)	0.130 (10)	−0.021 (6)	−0.031 (7)	0.076 (8)
C9	0.0179 (2)	0.0152 (2)	0.0194 (2)	−0.00212 (18)	0.00223 (18)	−0.00130 (17)
C10	0.0198 (2)	0.0212 (3)	0.0255 (2)	−0.0049 (2)	0.0040 (2)	−0.0005 (2)
H10	0.037 (5)	0.040 (5)	0.079 (7)	−0.005 (4)	0.021 (5)	−0.008 (5)
C11	0.0314 (3)	0.0213 (3)	0.0393 (3)	−0.0103 (2)	0.0014 (3)	0.0026 (2)
H11a	0.074 (6)	0.056 (6)	0.083 (8)	−0.008 (5)	0.036 (6)	0.005 (6)
H11b	0.043 (5)	0.045 (6)	0.075 (7)	−0.008 (4)	−0.020 (5)	−0.019 (5)
C12	0.0326 (3)	0.0156 (2)	0.0369 (3)	0.0006 (2)	−0.0068 (3)	−0.0028 (2)
H12a	0.084 (7)	0.052 (6)	0.054 (6)	−0.001 (5)	0.010 (6)	−0.026 (5)
H12b	0.076 (7)	0.036 (6)	0.095 (7)	−0.002 (5)	−0.044 (6)	0.014 (5)
C13	0.0190 (2)	0.0170 (2)	0.0186 (2)	−0.00053 (17)	0.00679 (18)	−0.00205 (18)
C14	0.0191 (2)	0.0211 (2)	0.0266 (3)	0.00204 (19)	0.0048 (2)	−0.0030 (2)
H14	0.042 (5)	0.069 (6)	0.037 (5)	0.016 (4)	−0.004 (4)	−0.012 (4)
C15	0.0206 (2)	0.0217 (3)	0.0461 (4)	0.0013 (2)	0.0121 (2)	−0.0016 (2)
H15	0.013 (4)	0.073 (7)	0.094 (8)	0.006 (4)	−0.004 (5)	−0.009 (6)
C16	0.0336 (3)	0.0255 (3)	0.0433 (4)	0.0037 (2)	0.0236 (3)	0.0013 (3)
C17	0.0467 (3)	0.0336 (3)	0.0258 (3)	0.0093 (3)	0.0181 (3)	−0.0035 (3)
H17	0.107 (8)	0.073 (7)	0.042 (6)	0.033 (6)	0.027 (6)	−0.017 (5)
C18	0.0320 (3)	0.0272 (3)	0.0194 (2)	0.0045 (2)	0.0062 (2)	−0.0053 (2)
H2	0.092 (7)	0.036 (5)	0.045 (6)	−0.037 (5)	0.027 (5)	−0.019 (4)
H18	0.061 (6)	0.057 (6)	0.038 (5)	0.018 (5)	−0.013 (5)	−0.020 (4)

### (2HAR) Geometric parameters (Å, º)

**Table:**

Si1—C1	1.8643 (5)	C16—C17	1.3844 (10)
Si1—C5	1.8646 (5)	C17—C18	1.3971 (8)
Si1—C9	1.8680 (5)	C2—H2	1.079 (7)
Si1—C13	1.8672 (5)	C3—H3a	1.082 (8)
O1—C1	1.3830 (6)	C3—H3b	1.067 (9)
O1—C4	1.4479 (6)	C4—H4a	1.039 (9)
O2—C5	1.3804 (6)	C4—H4b	1.093 (9)
O2—C8	1.4481 (7)	C6—H6	1.077 (7)
O3—C9	1.3847 (6)	C7—H7a	1.080 (9)
O3—C12	1.4596 (7)	C7—H7b	1.083 (8)
C1—C2	1.3350 (7)	C8—H8a	1.081 (9)
C2—C3	1.5081 (7)	C8—H8b	1.085 (10)
C3—C4	1.5272 (8)	C10—H10	1.049 (8)
C5—C6	1.3348 (7)	C11—H11a	1.072 (9)
C6—C7	1.5069 (8)	C11—H11b	1.102 (8)
C7—C8	1.5222 (8)	C12—H12a	1.085 (8)
C9—C10	1.3356 (7)	C12—H12b	1.072 (9)
C10—C11	1.5052 (8)	C14—H14	1.089 (8)
C11—C12	1.5271 (9)	C15—H15	1.092 (8)
C13—C14	1.4025 (7)	C16—H16	1.089 (7)
C13—C18	1.3975 (7)	C17—H17	1.083 (9)
C14—C15	1.3929 (7)	C18—H18	1.076 (8)
C15—C16	1.3851 (9)		
			
Si1—C1—O1	118.36 (3)	C1—C2—H2	124.0 (4)
Si1—C5—O2	118.53 (3)	C2—C3—H3a	114.0 (5)
Si1—C9—O3	119.68 (3)	C2—C3—H3b	111.4 (5)
Si1—C1—C2	128.55 (4)	C3—C2—H2	126.2 (4)
Si1—C5—C6	128.62 (4)	C3—C4—H4a	114.9 (6)
Si1—C9—C10	127.36 (4)	C3—C4—H4b	110.4 (5)
Si1—C13—C14	120.58 (4)	C4—C3—H3a	110.4 (5)
Si1—C13—C18	121.50 (4)	C4—C3—H3b	110.8 (6)
O1—C1—C2	113.09 (4)	C5—C6—H6	123.9 (4)
O1—C4—C3	107.11 (4)	C6—C7—H7a	111.6 (6)
O2—C5—C6	112.81 (5)	C6—C7—H7b	111.3 (5)
O2—C8—C7	107.48 (5)	C7—C6—H6	125.9 (4)
O3—C9—C10	112.96 (5)	C7—C8—H8a	111.7 (6)
O3—C12—C11	107.10 (5)	C7—C8—H8b	113.6 (6)
O1—C4—H4a	108.4 (5)	C8—C7—H7a	113.1 (5)
O1—C4—H4b	107.4 (5)	C8—C7—H7b	112.6 (5)
O2—C8—H8a	107.3 (5)	C9—C10—H10	125.0 (4)
O2—C8—H8b	108.1 (6)	C10—C11—H11a	110.4 (5)
O3—C12—H12a	106.4 (5)	C10—C11—H11b	111.6 (5)
O3—C12—H12b	108.1 (5)	C11—C10—H10	124.7 (4)
C1—Si1—C5	107.29 (2)	C11—C12—H12a	113.7 (5)
C1—Si1—C9	108.03 (2)	C11—C12—H12b	111.2 (6)
C1—Si1—C13	112.96 (2)	C12—C11—H11a	111.9 (5)
C5—Si1—C9	112.08 (2)	C12—C11—H11b	112.0 (5)
C5—Si1—C13	109.47 (2)	C13—C14—H14	119.9 (4)
C9—Si1—C13	107.08 (2)	C13—C18—H18	118.9 (4)
C1—O1—C4	107.51 (4)	C14—C15—H15	120.1 (5)
C5—O2—C8	107.87 (4)	C15—C14—H14	118.8 (4)
C9—O3—C12	107.43 (4)	C15—C16—H16	119.9 (6)
C1—C2—C3	109.84 (5)	C16—C15—H15	119.7 (5)
C2—C3—C4	101.48 (4)	C16—C17—H17	120.9 (5)
C5—C6—C7	110.24 (5)	C17—C16—H16	120.8 (6)
C6—C7—C8	101.58 (5)	C17—C18—H18	120.6 (4)
C9—C10—C11	110.34 (5)	C18—C17—H17	118.2 (5)
C10—C11—C12	101.65 (5)	H3a—C3—H3b	108.7 (7)
C13—C14—C15	121.27 (5)	H4a—C4—H4b	108.3 (8)
C13—C18—C17	120.48 (6)	H7a—C7—H7b	106.8 (7)
C14—C13—C18	117.88 (5)	H8a—C8—H8b	108.4 (10)
C14—C15—C16	120.16 (6)	H11a—C11—H11b	109.2 (7)
C15—C16—C17	119.30 (5)	H12a—C12—H12b	110.1 (7)
C16—C17—C18	120.89 (5)		

### Å

**Table:**
